# Theranostic Potential of an NAD(P)H‐Activatable Fluorophore: Assessing Cancer Aggressiveness and Triggering Apoptosis

**DOI:** 10.1002/anie.7968058

**Published:** 2026-08-25

**Authors:** Yujin Cha, Taehoon Oh, Hui Joo Kim, Minju Kang, Shin A Yoon, Yul Lee, Yeon‐Ju Lee, Sun Young Park, Dong‐Sik Shin, Sung‐Kyun Ko, Jiyou Han, Min Hee Lee

**Affiliations:** ^1^ Department of Chemistry Sookmyung Women's University Seoul Republic of Korea; ^2^ Chemical Biology Research Center Korea Research Institute of Bioscience and Biotechnology (KRIBB) Cheongju Republic of Korea; ^3^ College of Pharmacy Chungbuk National University Cheongju Republic of Korea; ^4^ Department of Chemistry Chung‐Ang University Seoul Republic of Korea; ^5^ Therapeutics & Biotechnology Division Korea Research Institute of Chemical Technology (KRICT) Daejeon South Korea; ^6^ Department of Chemical and Biological Engineering Sookmyung Women's University Seoul Republic of Korea; ^7^ KRIBB School of Bioscience University of Science and Technology (UST) Daejeon Republic of Korea; ^8^ Department of Biomedical and Chemical Sciences, Laboratory of Stem Cell Research and Biotechnology Hyupsung University Hwaseong‐si Republic of Korea

**Keywords:** apoptosis, cancer aggressiveness, fluorescence imaging, NAD(P)H, theranostics

## Abstract

Molecular theranostics, integrating precise diagnosis with targeted therapy, represents a cornerstone of personalized medicine. As a pivotal metabolic cofactor, reduced nicotinamide adenine dinucleotide (phosphate) (NAD(P)H) is intrinsically linked to cancer progression and survival when dysregulated. In this study, we report **DE‐CQ**, an NAD(P)H‐activatable fluorophore that enables both cancer imaging and apoptosis. **DE‐CQ** allows the real‐time quantification and visualization of intracellular NAD(P)H, serving as a robust molecular indicator of cancer malignancy. Beyond its diagnostic utility, **DE‐CQ** preferentially induces mitochondria‐ and ER stress‐dependent, caspase‐mediated apoptosis in aggressive MDA‐MB‐231 cancer cells while maintaining minimal toxicity toward normal cells. Our findings demonstrate that **DE‐CQ** functions as a potent theranostic agent, simultaneously providing diagnostic insights into cancer aggressiveness and triggering cell death. Given its dual functionality and high selectivity, **DE‐CQ** holds significant potential as a next‐generation platform for advanced, personalized cancer treatment.

## Graphical abstract

**DE‐CQ**, an NAD(P)H‐activatable theranostic probe, enables the quantification of cancer aggressiveness via glucose‐stimulated fluorescence imaging and selectively induces apoptosis. Its multifaceted efficacy is demonstrated across 2D cells, 3D spheroids, and in vivo models.

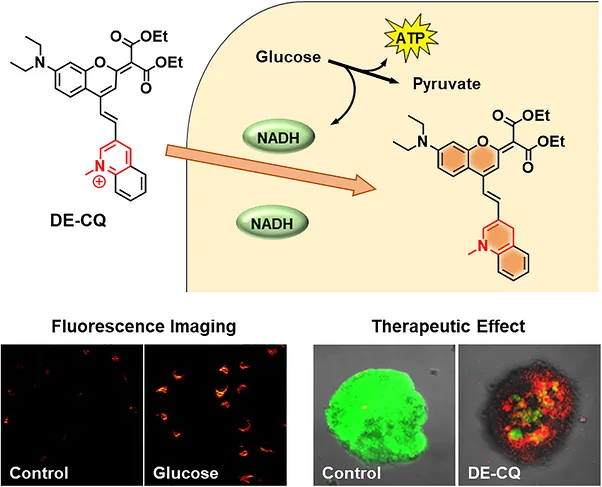

## 1 Introduction

Theranostics integrates diagnostic imaging with targeted drug delivery, providing a promising platform for personalized medicine [1, 2, 3, 4, 5, 6, 7]. Conventional theranostic systems typically combine a targeting ligand, prodrug, fluorophore, and cleavable linker to enable selective accumulation in diseased tissues, controlled drug release, and fluorescence activation in response to specific enzymes or metabolites. Such systems can reduce off‐target toxicity through localized prodrug activation, and diverse theranostic approaches have therefore been developed for various diseases [8, 9, 10]. However, their construction often requires complex multicomponent conjugation. The development of simpler theranostic motifs and the identification of disease‐relevant biomolecular targets are therefore important for practical applications.

Reduced nicotinamide adenine (phosphate) dinucleotide, NAD(P)H, is a key redox cofactor involved in cellular proliferation, aging, and apoptosis [11, 12, 13, 14, 15]. Disruption of NAD(P)H/NAD(P)^+^ balance is associated with various pathological conditions, including diabetes, neurodegenerative disease, and cancer [16, 17, 18, 19]. In particular, elevated NAD(P)H production is closely associated with aggressive cancer cell proliferation [20, 21, 22]. Even in the presence of oxygen, cancer cells preferentially rely on aerobic glycolysis, known as the Warburg effect, to support their metabolic demands [23, 24, 25, 26]. This metabolic reprogramming alters the intracellular NAD(P)H pool and contributes to redox homeostasis and cancer progression. Mitochondrial respiration remains under these conditions and can generate reactive oxygen species (ROS), which influence apoptosis and migration [27, 28, 29, 30, 31]. To counteract oxidative stress, cancer cells maintain elevated NAD(P)H levels to provide reducing equivalents for glutathione recycling and cellular survival [32, 33, 34]. Accordingly, elevated NAD(P)H is both an indicator of cancer aggressiveness and a promising target for cancer diagnosis and therapy.

The biological importance of NAD(P)H has stimulated considerable interest in monitoring its levels in living cells and tissues, primarily using NAD(P)H‐selective fluorophores [35, 36, 37, 38, 39, 40, 41, 42, 43]. Such imaging can provide valuable insights into cancer metabolism and support the development of therapeutic strategies. Recent NAD(P)H‐responsive drug delivery systems have progressed from enzyme‐dependent quinone triggers, including NQO1‐responsive systems, to enzyme‐free, pyridinium‐ and quinolinium‐based units that undergo direct hydride reduction [35]. Nevertheless, most reported systems still rely on complex multicomponent conjugation to integrate sensing and therapeutic functions.

Here, we report **DE‐CQ**, an NAD(P)H‐activatable fluorophore that functions as a potential theranostic agent for cancer imaging and apoptosis induction (Scheme 1). **DE‐CQ** contains a coumarin‐fused quinolinium scaffold in which the quinolinium moiety acts as both an NAD(P)H‐responsive unit and a fluorescence switch. Reduction by NAD(P)H produces a large Stokes shift of more than 110 nm, enabling high‐contrast fluorescence imaging. In addition to its sensing function, the coumarin‐based scaffold provides pharmacological potential, as appropriate structural modification can promote apoptosis and transform a conventional imaging probe into a functional theranostic agent [44]. We evaluated the fluorescence response, cytotoxicity, and mechanism of action of **DE‐CQ** in several cancer cell lines, including highly aggressive MDA‐MB‐231 cells. **DE‐CQ** further exhibited theranostic activity in 3D tumor spheroids and an in vivo xenograft model. Compared with previously reported NAD(P)H‐sensitive probes (Table S1), **DE‐CQ** combines high diagnostic sensitivity and apoptosis‐mediated therapeutic activity, demonstrating its potential as a simple theranostic platform for aggressive cancers.

**SCHEME 1:**
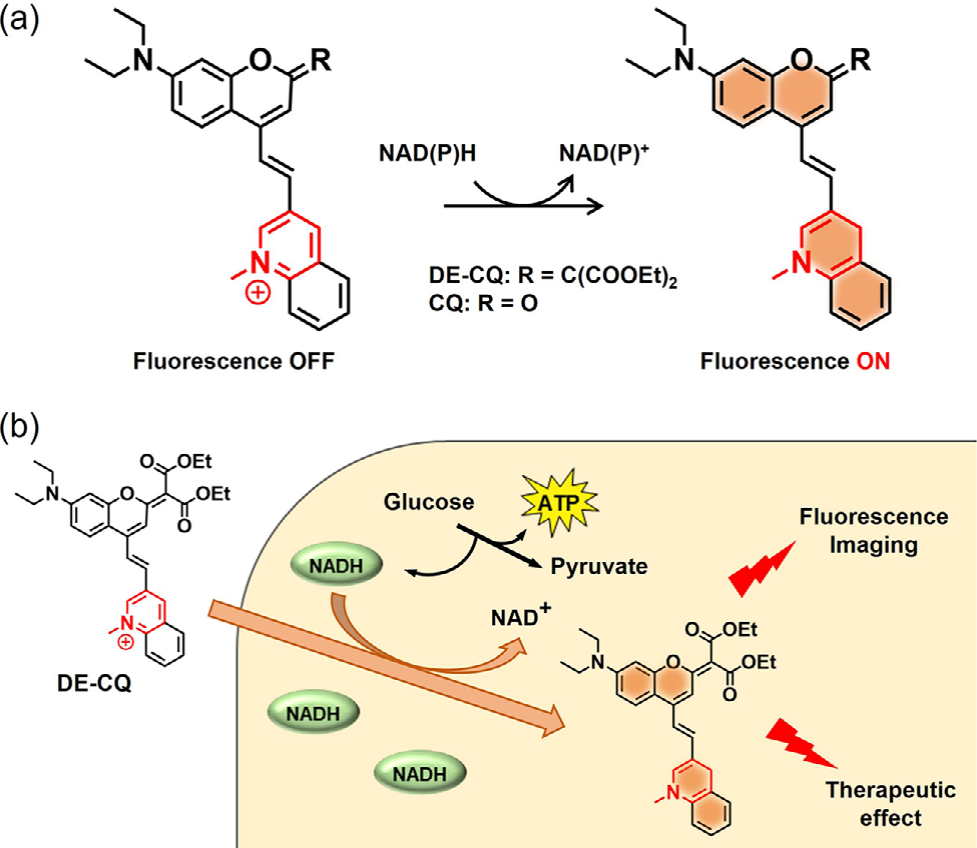
(a) DE‐CQ and CQ are sensitive to the reductive activity of NAD(P)H and act as fluorescence switches for NAD(P)H. (b) DE‐CQ indicates cancer aggressiveness and induces cancer apoptosis and is proposed as a fascinating potential theranostic agent.

## 2 Results and Discussion

The synthetic pathways for **DE‐CQ** and **CQ** are illustrated in Figure 1a. Briefly, to incorporate a diester group in the coumarin **3**, 7‐diethylamino‐4‐methylcoumarin was employed as a starting material. This was condensed with 3‐quinoline carboxaldehyde to yield the coumarin‐quinoline **1**. Similarly, compound **2** was synthesized from coumarin **3**. Subsequent methylation of **1** and **2** afforded the novel coumarin‐quinoliniums, **CQ** and **DE‐CQ**, respectively. Their chemical structures were convincingly confirmed by ^1^H and ^13^C NMR and mass spectrometry. Detailed spectra and analytical data are provided in the Supporting Information (Figures S1–S15).

**FIGURE 1:**
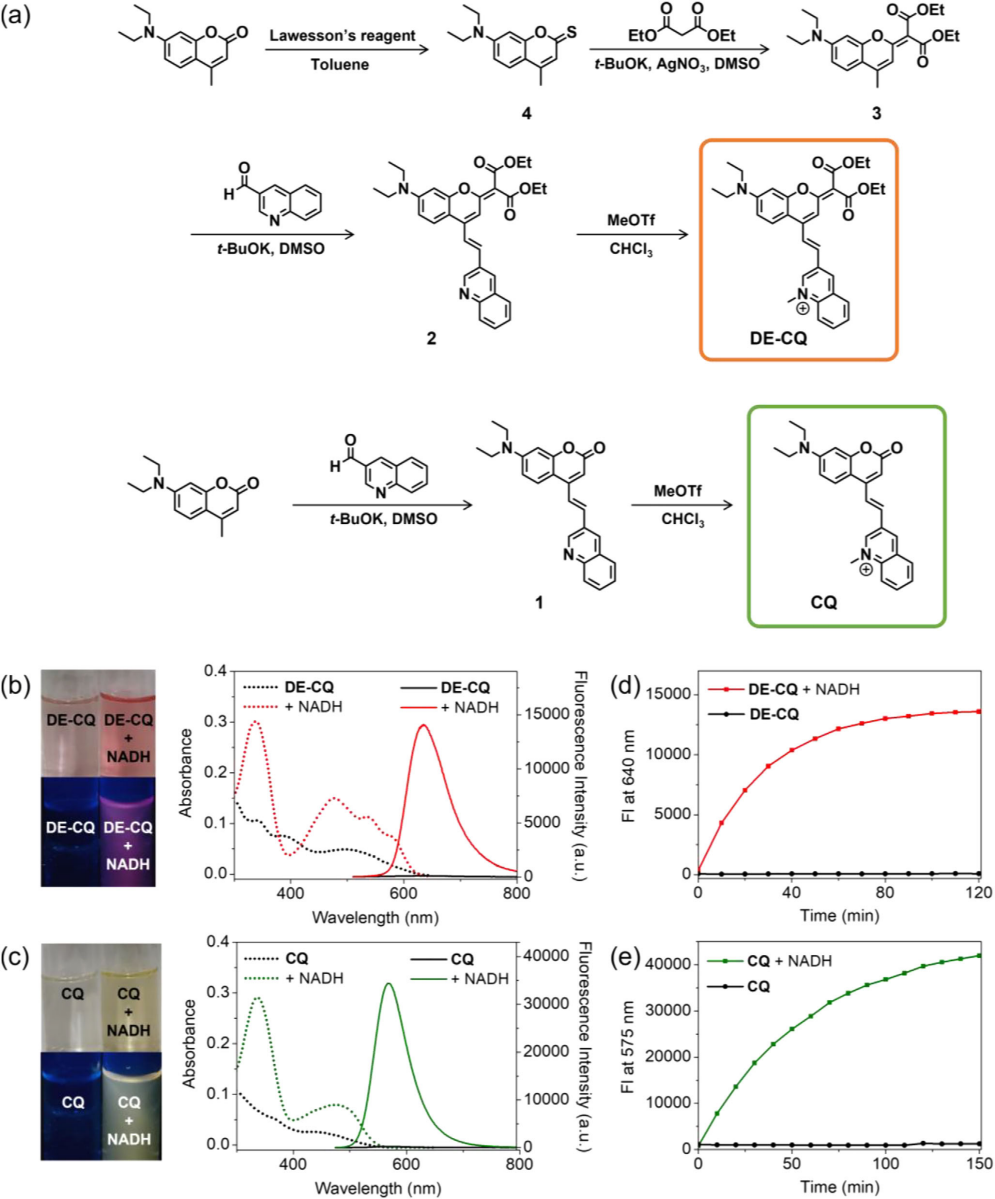
Synthesis of NAD(P)H‐activatable fluorophores and their fluorescence switch properties to NADH. (a) Synthetic pathways of DE‐CQ and CQ used in this study. (b, c) Photographs for their solution color and fluorescence before and after NADH addition and corresponding absorption and fluorescence spectra. (d, e) Kinetic curves of DE‐CQ and CQ (each 5 µM) for NADH activity (50 µM).

To evaluate the NADH sensing performance of **DE‐CQ** and **CQ** in aqueous media, their photophysical properties were investigated in PBS solution (pH 7.4, 10 mM, 37°C, containing 1 mM CTAB and 1% v/v DMSO). Initially, **DE‐CQ** displayed a broad absorption band centered near 500 nm with negligible fluorescence, while **CQ** showed an absorption maximum at 465 nm and was similarly nonfluorescent (Figure 1b,c). Upon the addition of NADH, both probes exhibited distinct color shifts (pale red to deep red for **DE‐CQ**; pale yellow to deep yellow for **CQ**) accompanied by robust fluorescence enhancements at 640 nm and 575 nm, respectively.

The fluorescence intensities of both probes increased proportionally with NADH concentration and incubation time (Figures 1d,e and S16). Kinetic analysis of the time‐dependent spectral changes yielded observed rate constants (*k_obs_*) of 5.70 × 10^−4^ s^−1^ for **DE‐CQ** and 2.78 × 10^−4^ s^−1^ for **CQ**, confirming a faster reduction rate for **DE‐CQ**. Furthermore, time‐resolved fluorescence measurements and quantum yield (Φ*_f_*) calculations revealed that NADH‐mediated reduction significantly increases the radiative decay rate constant (*k_r_*), providing numerical proof for the observed fluorescence turn‐on response (Figure S17 and Table S2). The reduction process was confirmed to be irreversible, as the fluorescence of the reduced probes remained stable upon NAD^+^ addition over 6 h (Figure S18). Notably, linear calibration curves (0–10 µM) yielded limits of detection (LOD) of 0.038 µM for **DE‐CQ** and 0.019 µM for **CQ** (Figure S19). These results demonstrate that the fluorescence wavelength is tunable, and the sensitivity is significantly improved compared to our previous reports [39].

As outlined in Scheme 1, this fluorescence activation is attributed to an NADH‐triggered modulation of the internal charge transfer (ICT) process [45], where reduction of the electron‐deficient quinolinium moiety into an electron‐rich quinoline unit restores the ICT pathway. DFT calculations were performed on the probes before and after NADH reduction (Figure S20). Upon reduction, a decreased HOMO–LUMO energy gap and an increased oscillator strength (*f*) confirm the enhanced ICT character and the fluorescence turn‐on mechanism.

In addition, we investigated the effect of pH on the NADH‐mediated fluorescence activation of **DE‐CQ** and **CQ**, which is particularly beneficial for biological assays. Both probes exhibited remarkable fluorescence intensities with superior signal‐to‐noise ratios in the presence of NADH across a broad biological pH range (pH 4–8) (Figure 2a,d). This suggests that **DE‐CQ** and **CQ** can provide reliable NADH detection regardless of pH variations within different cellular compartments. Furthermore, we evaluated the selectivity of the probes against various biological interferants, including metal ions, anions, ROS, thiols, glucose, adenosine triphosphate (ATP), adenosine diphosphate (ADP), and nicotinamide adenine dinucleotide (NAD^+^). Significant fluorescence was exclusively observed in the presence of NADH and NADPH (Figure 2b,e), confirming that **DE‐CQ** and **CQ** function as highly specific fluorescent “Off‐On” switches for reduced nicotinamide cofactors.

**FIGURE 2:**
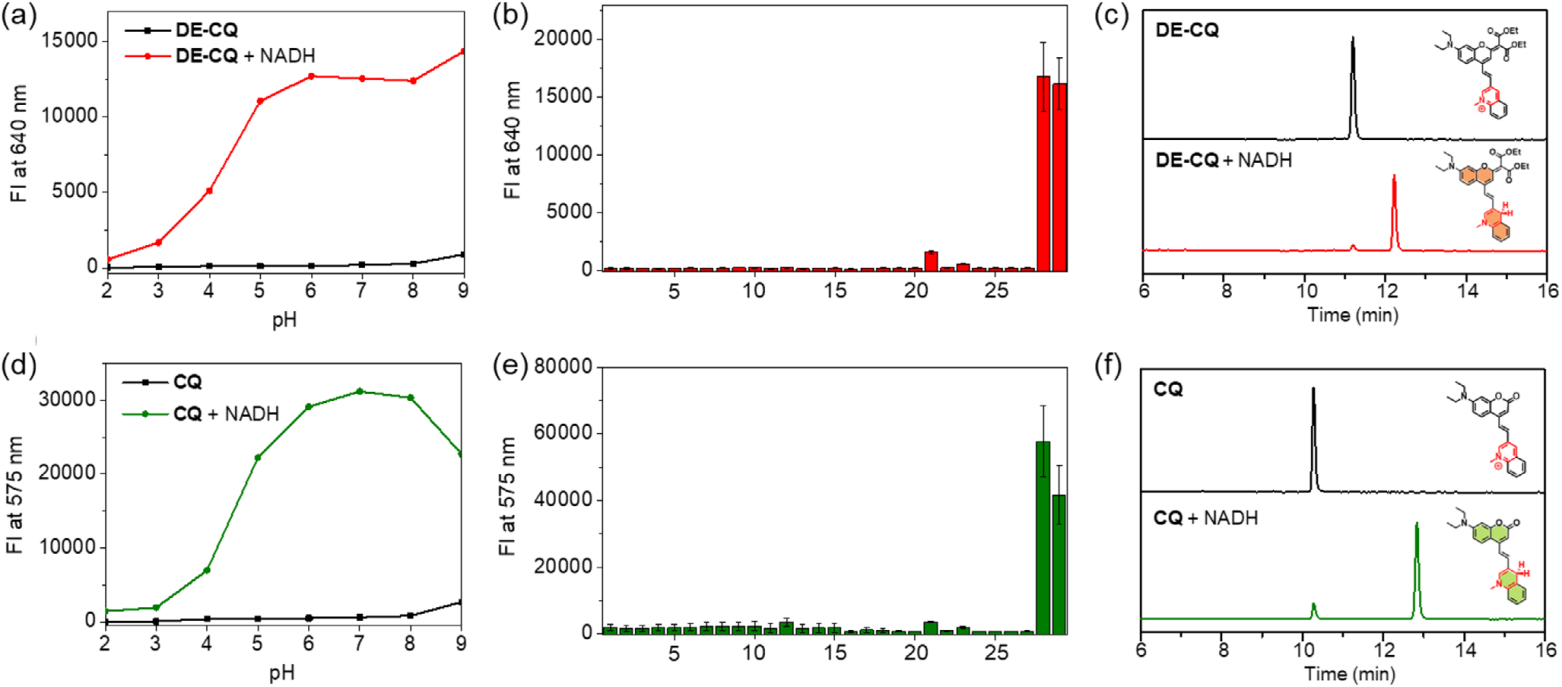
Fluorescence activation of DE‐CQ and CQ by NAD(P)H and analysis using liquid chromatography and mass spectrometry. (a, d) Fluorescence intensities of DE‐CQ and CQ before and after NAD(P)H addition in different pH solutions. (b, e) Fluorescence response to various biocomponents (100 µM, respectively; 1 mM for GSH and glucose), (1) DE‐CQ or CQ, (2) K^+^, (3) Na^+^, (4) Cu^+^, (5) Cu^2+^, (6) Ca^2+^, (7) Mg^2+^, (8) Zn^2+^, (9) Fe^2+^, (10) Fe^3+^, (11) Cl^−^, (12) CN^−^, (13) H_2_PO_4_^−^, (14) OAc^−^, (15) OH^−^, (16) H_2_O_2_, (17) HOO^t^Bu, (18) ·O_2_^−^, (19) ·OH, (20) ·O^t^Bu, (21) GSH, (22) Cys, (23) Hcy, (24) glucose, (25) ATP, (26) ADP, (27) NAD^+^, (28) NADPH, (29) NADH. All fluorescence data were recorded after 80 min incubation at 37°C in a PBS solution (pH 7.4, 10 mM) containing 1 mM CTAB and 1% (v/v) DMSO. Excitations were respectively applied at 490 nm for DE‐CQ and 465 nm for CQ. (c, f) LC profiles before and after the NAD(P)H reaction. LC peaks were shown by detection of absorbance for DE‐CQ and CQ.

To validate the reductive transformation, the reaction products were rigorously analyzed using HPLC and mass spectrometry. Initially, **DE‐CQ** showed a distinct LC peak at 11.2 min with a mass of 527.2571 m/z ([M]^+^). Upon incubation with NADH, a new peak emerged at 12.2 min with an m/z of 529.58, corresponding to the reduced form ([M+H]^+^) (Figures 2c and S21). Similar reductive behavior was clearly observed for **CQ** (Figures 2f and S21), confirming that the NADH‐mediated reduction of the quinolinium moiety is the key driver of fluorescence activation, as proposed in Scheme 1.

We subsequently investigated whether the NAD(P)H‐sensing capabilities of **DE‐CQ** and **CQ** could be leveraged for cancer imaging in live cells using fluorescence microscopy. In preliminary evaluations using MDA‐MB‐231 cancer cells, both probes demonstrated excellent cell permeability, resulting in detectable intracellular fluorescence (Figure 3a; Figures S22 and S23). Notably, the fluorescence intensity increased progressively in a time‐ and concentration‐dependent manner. Furthermore, the nonfluorescent nature of the unreacted probes facilitated real‐time monitoring without the need for additional washing steps, thereby minimizing experimental artifacts and enabling more accurate visualization of cellular NAD(P)H dynamics.

**FIGURE 3:**
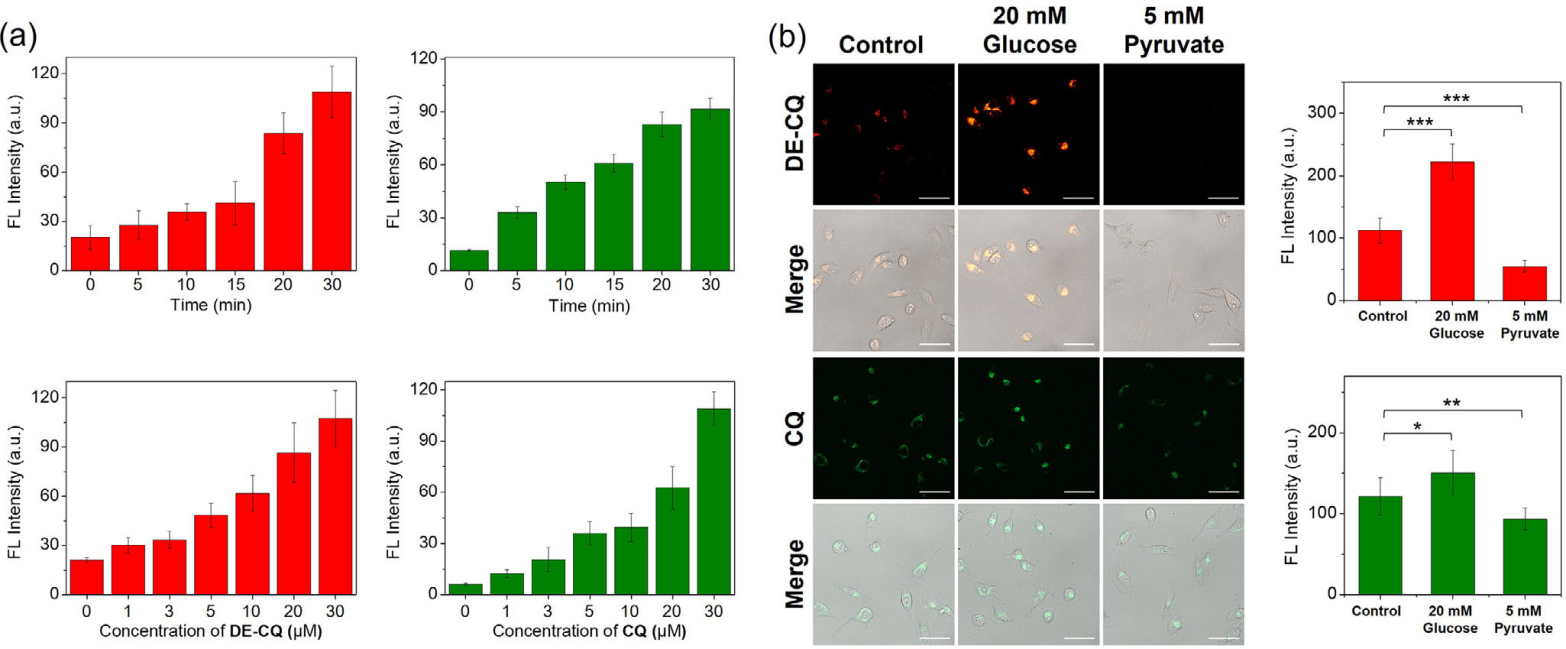
Cell permeability and fluorescence intensity of DE‐CQ and CQ in live cells. (a) Time‐dependent and dose‐dependent fluorescence intensities of MDA‐MB‐231 cells treated with DE‐CQ or CQ. (b) Fluorescence imaging of cells under glycolysis stimuli. Cells were pretreated with 20 mM glucose or 5 mM pyruvate, and then incubated with 10 µM DE‐CQ or 10 µM CQ for 15 min. All fluorescence images were collected by the excitation and emission filter sets; *λ*_ex_: 540–552 nm and *λ*_em_: 575–640 nm for DE‐CQ or *λ*_ex_: 450–490 nm and *λ*_em_: 500–550 nm for CQ. The bottom image is an overlay of the fluorescent and nonfluorescent phase contrast images. Scale bar: 50 µm. *p < 0.05, **p < 0.01, and ***p < 0.001.

To confirm whether the fluorescence fluctuations of **DE‐CQ** and **CQ** directly linked to changes in intracellular NAD(P)H levels, we modulated cellular glycolysis using glucose and pyruvate treatments. In cellular metabolism, excess glucose stimulates glycolysis to enhance NAD(P)H production, whereas pyruvate treatment consumes the NAD(P)H pool through lactic acid fermentation [46]. To avoid starvation‐induced metabolic artifacts such as autophagy, a standard nutrient‐rich medium was maintained as the control group, rather than a glucose‐free medium. We previously validated that treatment with an additional 20 mM glucose significantly increases the intracellular NAD(P)H concentration in MDA‐MB‐231 cells [39, 43, 47]. Upon treating these cells with 10 µM of **DE‐CQ** or **CQ** for 15 min, fluorescence microscopy revealed distinct intensity variations. Glucose‐pretreated cells exhibited enhanced fluorescence, while pyruvate‐pretreated cells showed a marked decrease compared to the untreated control group (Figure 3b). These results substantiate that the fluorescence of both probes is primarily activated by NAD(P)H. Notably, **DE‐CQ** demonstrated a more pronounced response to these metabolic stimuli than **CQ**, indicating its superior sensitivity to glycolysis‐induced NAD(P)H production. Consequently, we selected **DE‐CQ** as the optimal NAD(P)H‐activatable fluorophore for subsequent cancer imaging studies.

The upregulation of glycolysis is a well‐established hallmark of cancer, leading to the characteristic overproduction of NAD(P)H [17, 21, 27]. These metabolic features are particularly prominent in aggressive cancers characterized by high metastasis rates and poor prognosis. We anticipated that **DE‐CQ** would be effectively activated in such aggressive environments, yielding robust fluorescence signals. Furthermore, we envisioned that by comparing cellular fluorescence intensities before and after glucose stimulation, we could assess the metabolic “aggressiveness” of individual cancer cells based on their NAD(P)H turnover. This diagnostic approach could serve as a valuable tool for determining cancer aggressiveness and providing critical insights into cancer prognosis.

Based on this concept, we evaluated the fluorescence response of **DE‐CQ** toward glucose treatment across various cancer cell lines derived from different tissues. To compensate for the varying basal NAD(P)H levels and probe uptake efficiencies across diverse cell lines, the metabolic aggressiveness of each cell line was evaluated based on the relative fluorescence response (fold change and difference) rather than absolute intensities. The intracellular fluorescence intensity was recorded and quantified following treatment with 20 mM glucose, as summarized in Figure 4. Significant increases in fluorescence were observed in specific cell lines, including breast (MDA‐MB‐231, T47D), cervical (HeLa), pancreatic (PANC‐1), prostate (LNCaP‐LN3), gastric (AGS), and colon (HCT‐116) cancer cells (Figure 4a,b). In contrast, other cell lines, such as breast (MCF‐7), lung (A549), prostate (DU‐145), and liver (HepG2, HuH‐7) cancer cells, exhibited only negligible changes or slight decreases in fluorescence intensity (Figures S24 and ‐S25).

**FIGURE 4:**
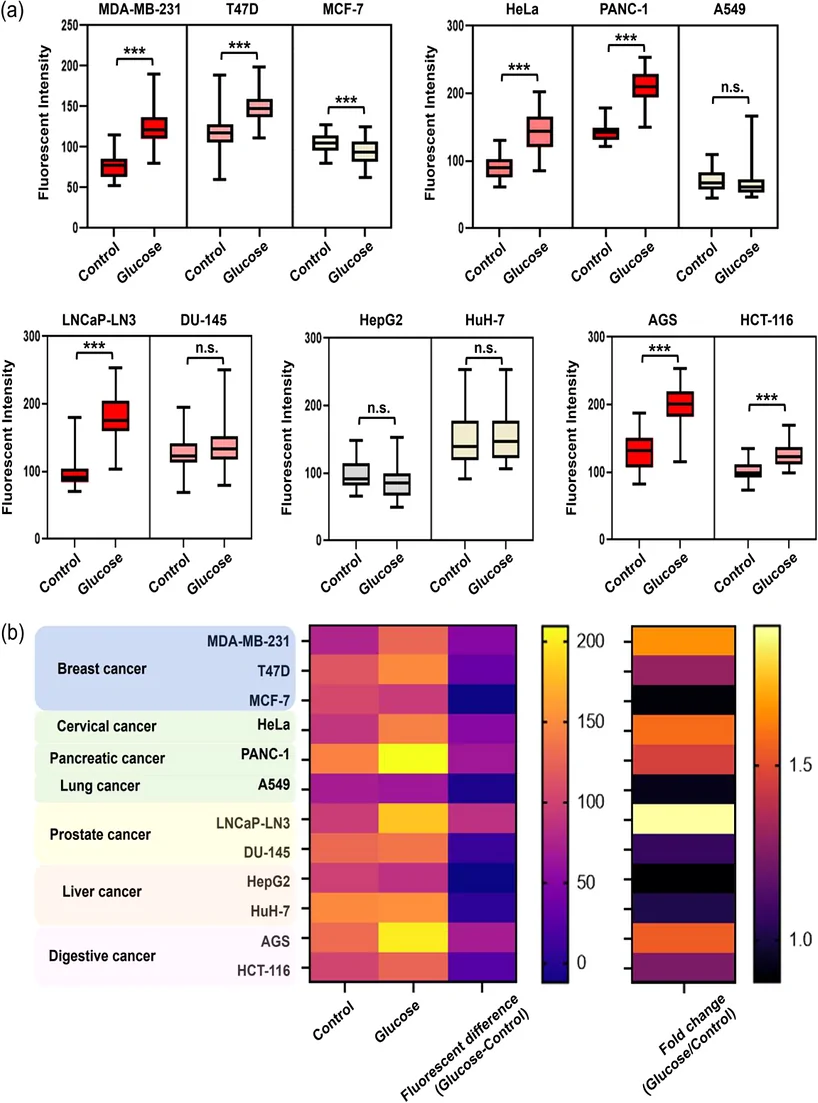
Fluorescence responses of various cancer cell lines before and after glucose stimulation using DE‐CQ. (a) Box plots of quantified fluorescence intensities of each cell line. ***p < 0.001, n.s.: not significant. (b) Heat map analysis of fluorescent difference (glucose‐control) and fold change (glucose/control). Cells were treated with 20 mM glucose for 45 min, and then incubated with 10 µM DE‐CQ for 15 min. All data were derived from fluorescence images collected with the excitation and emission filter set as *λ*_ex_: 540–552 nm and *λ*_em_: 575–640 nm. All images were acquired and processed under identical instrumental settings for accurate quantitative comparison.

The pronounced fluorescence enhancement observed in MDA‐MB‐231 cells correlates with elevated NAD(P)H levels stemming from upregulated glucose metabolism, a hallmark of aggressive cancers with high metastatic potential and poor prognoses [48, 49, 50, 51, 52, 53, 54, 55]. In contrast, other cancer cell lines, such as A549, DU‐145, and HepG2, exhibited negligible metabolic responses (cf. Figure S25). Notably, within breast cancer subtypes, the degree of fluorescence activation followed the order of MDA‐MB‐231 and > T47D > MCF‐7, directly mirroring their relative aggressiveness. This trend was further validated by heatmap analysis and aligns with the recognized status of MDA‐MB‐231 as a representative model of highly malignant phenotypes [56, 57]. The versatility of **DE‐CQ** in assessing malignancy was also confirmed in prostate cancer models, where the more aggressive LNCaP‐LN3 line exhibited a significant fluorescence increase upon glucose treatment compared to the less responsive DU‐145 [52, 58]. Collectively, these results demonstrate that **DE‐CQ** provides a robust, visible, and quantitative platform for monitoring intracellular NAD(P)H, effectively serving as a metabolic indicator of cancer aggressiveness.

Next, we evaluated the theranostic efficiency of **DE‐CQ** by investigating its cytotoxic effects via MTT assay in MDA‐MB‐231 breast cancer cells and MCF10A normal breast cells. Notably, after 24 or 48 h of incubation with 10 µM **DE‐CQ**, MDA‐MB‐231 cells exhibited above 50% cell death, whereas the normal cell line, MCF10A, showed relatively low cytotoxicity (Figure S26). In contrast, **CQ** showed minimal cytotoxicity in both MDA‐MB‐231 and MCF10A cells. These results strongly suggest that **DE‐CQ** can act as a potential anticancer therapeutic agent, preferentially inducing apoptosis in cancer cells over normal cells.

To further elucidate the underlying mechanism, we performed real‐time PCR analysis in MDA‐MB‐231 cells. The results revealed that **DE‐CQ** treatment leads to the upregulation of mitochondria‐mediated apoptotic genes (e.g., *Fas*, *Bax*, *Caspase‐9*, and *Caspase‐3*) and endoplasmic reticulum (ER) stress‐mediated apoptotic genes (*PERK*, *CHOP*, *JNK*, and *Bim*) (Figure 5a,b and Table S3).

**FIGURE 5:**
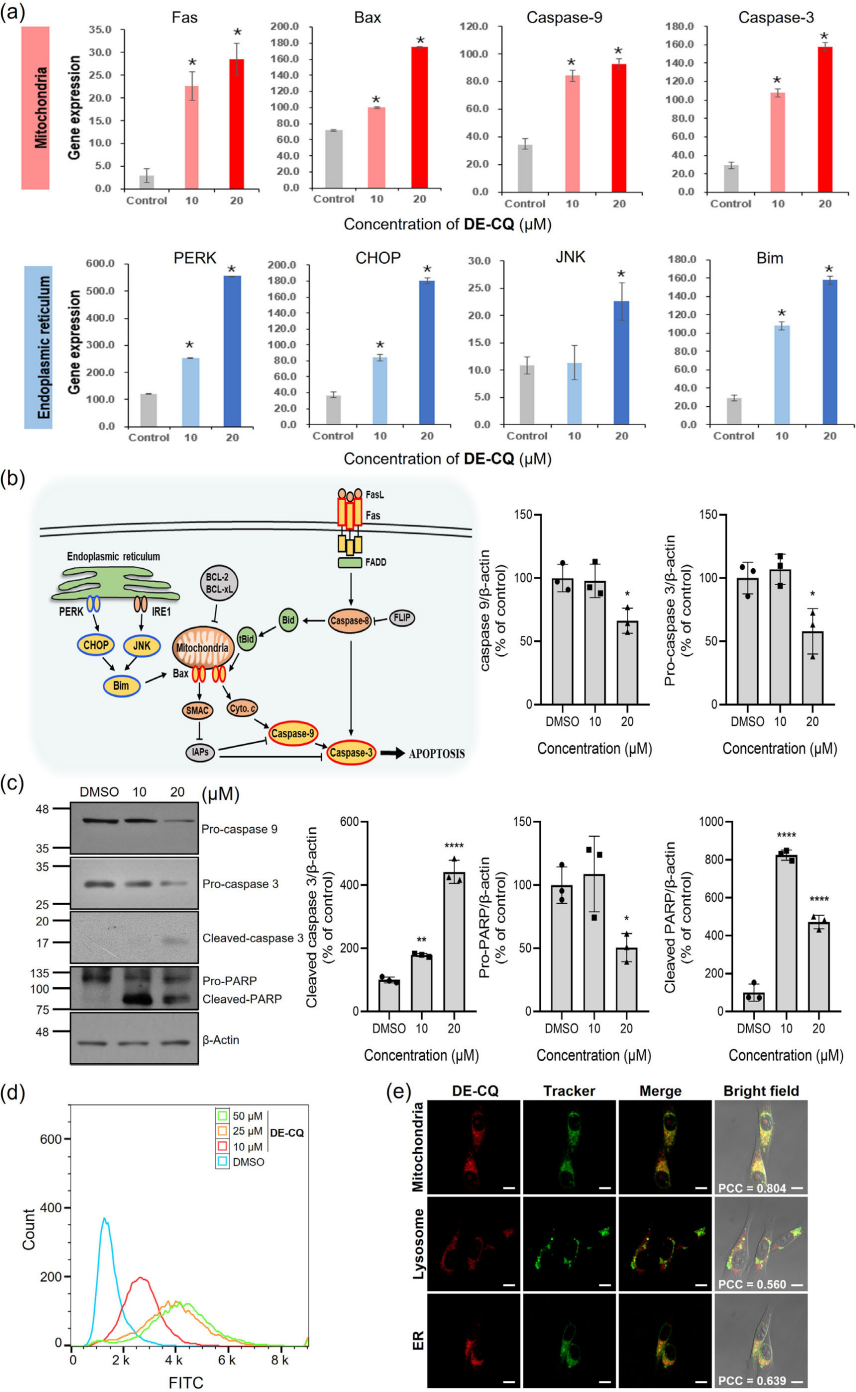
Apoptosis mode and intracellular localization of active DE‐CQ in live cells. (a) Apoptotic gene expression in MDA‐MB‐231 cells treated with DE‐CQ (0, 10, or 20 µM) for 24 h determined by quantitative PCR analysis. *p < 0.05. (b) Schematic illustration of the mitochondrial and endoplasmic reticulum (ER) caspase‐dependent apoptotic pathways. (c) Representative Western blot analysis and densitometric quantification of pro‐caspase‐9, pro‐caspase‐3, cleaved caspase‐3, full‐length PARP, and cleaved PARP in MDA‐MB‐231 cells treated with DE‐CQ (0, 10, or 20 µM) for 48 h. (d) MDA‐MB‐231 cells were treated with DE‐CQ (0, 10, 25, or 50 µM) for 24 h, stained with Annexin V‐FITC, and analyzed by flow cytometry. (e) Intracellular fluorescence colocalization of DE‐CQ (red) with organelle‐specific trackers for mitochondria, ER, and lysosomes (green) evaluated by confocal fluorescence microscopy. Pearson correlation coefficient (PCC) indicates the degree of colocalization. All confocal fluorescence images were collected using a 488 nm excitation laser and 493–520 nm (green) and 644–700 nm (red) emission band‐pass filters. Scale bar: 10 µm. Data in (a) and (c) are presented as the mean ± SD (n = 3). *p < 0.05.

To further examine the cellular events associated with **DE‐CQ**‐induced apoptosis, intracellular ROS levels and mitochondrial membrane potential were assessed by flow cytometry. H_2_DCF‐DA fluorescence increased following **DE‐CQ** treatment in a time‐ and concentration‐dependent manner, whereas NAC co‐treatment partially attenuated this increase (Figure S27a,b). Consistently, **DE‐CQ** decreased DiOC_6_ fluorescence, suggesting mitochondrial membrane depolarization, and this change was partially reversed by NAC (Figure S27c). These observations further support the involvement of oxidative and mitochondrial stress in **DE‐CQ**‐induced cell death.

To further validate the activation of apoptotic signaling at the protein level, Western blot analysis was performed in MDA‐MB‐231 cells treated with **DE‐CQ**. Consistent with the transcriptional upregulation of apoptosis‐related genes, **DE‐CQ** treatment decreased the levels of pro‐caspase‐9, pro‐caspase‐3, and full‐length PARP, accompanied by increased levels of cleaved caspase‐3 and cleaved PARP (Figure 5c), supporting activation of caspase‐dependent apoptotic signaling. In addition, flow cytometric analysis using Annexin V‐FITC staining demonstrated a concentration‐dependent increase in Annexin V‐positive cells following **DE‐CQ** treatment (Figure 5d). Collectively, these findings indicate that **DE‐CQ** possesses theranostic capabilities by triggering the caspase‐dependent apoptotic pathway through both mitochondrial dysfunction and ER stress.

Intracellular fluorescence localization experiments, conducted using various organelle‐specific trackers under confocal microscopy, revealed that the fluorescence of **DE‐CQ** in MDA‐MB‐231 cells primarily co‐localizes with the mitochondria tracker (PCC value: 0.804). Significant colocalization was also observed with the ER tracker (PCC value: 0.639) and, to a lesser extent, the lysosome tracker (PCC value: 0.560) (Figure 5e). These findings are in excellent agreement with the cytotoxic mechanisms described above, confirming that **DE‐CQ** targets the mitochondria and ER to trigger organelle‐specific apoptotic pathways.

Furthermore, as shown in Figure S28, **DE‐CQ** treatment significantly downregulated the expression of stemness markers (e.g., *Nanog* and *CD133*) and epithelial‐mesenchymal transition (EMT) markers (*Snail1*, *E‐cadherin*, and *MMP9*), which are closely associated with cancer metastasis. This inhibitory effect was particularly pronounced in MDA‐MB‐231 cells. These results suggest that **DE‐CQ** may function as a potent therapeutic agent by modulating cancer cell stemness and attenuating the EMT process, thereby potentially suppressing the metastatic potential of aggressive cancer cells.

Consistent with these molecular changes, the effects of **DE‐CQ** on the migratory and invasive behavior of MDA‐MB‐231 cells were further examined. **DE‐CQ** increased the residual wound width in the scratch wound‐healing assay and reduced Matrigel‐dependent cell invasion in a concentration‐dependent manner (Figure S29a,b). Time‐lapse single‐cell tracking further showed restricted cell trajectories and reduced cumulative motility following **DE‐CQ** treatment (Figure S29c–e). Together, these findings support the inhibitory effect of **DE‐CQ** on the migratory and invasive phenotypes of MDA‐MB‐231 cells.

Then, we validated the theranostic efficacy of **DE‐CQ** in cancer tissue‐mimicking 3D spheroids using confocal fluorescence microscopy. MDA‐MB‐231 tumor spheroids were prepared to mimic key features of the breast tumor microenvironment. In the diagnostic imaging mode, spheroids pretreated with 20 mM glucose and incubated with 10 µM **DE‐CQ** exhibited significantly enhanced fluorescence intensity compared with untreated controls (Figure 6a,c). To evaluate its therapeutic potential, spheroids treated with 10 µM **DE‐CQ** for 12 h showed substantial cell death (Figure 6b,c). Live/dead cell imaging using Calcein AM (green) and propidium iodide (PI, red) further revealed extensive cell death throughout the spheroid (Figure 6d). Time‐dependent ATP imaging and viability analysis further revealed progressive reductions in ATP levels and spheroid viability following **DE‐CQ** treatment (Figure S30). Together, these results demonstrate that **DE‐CQ** penetrates dense 3D tumor structures, enables high‐contrast imaging, and induces a pronounced therapeutic response throughout the tumor spheroids.

**FIGURE 6:**
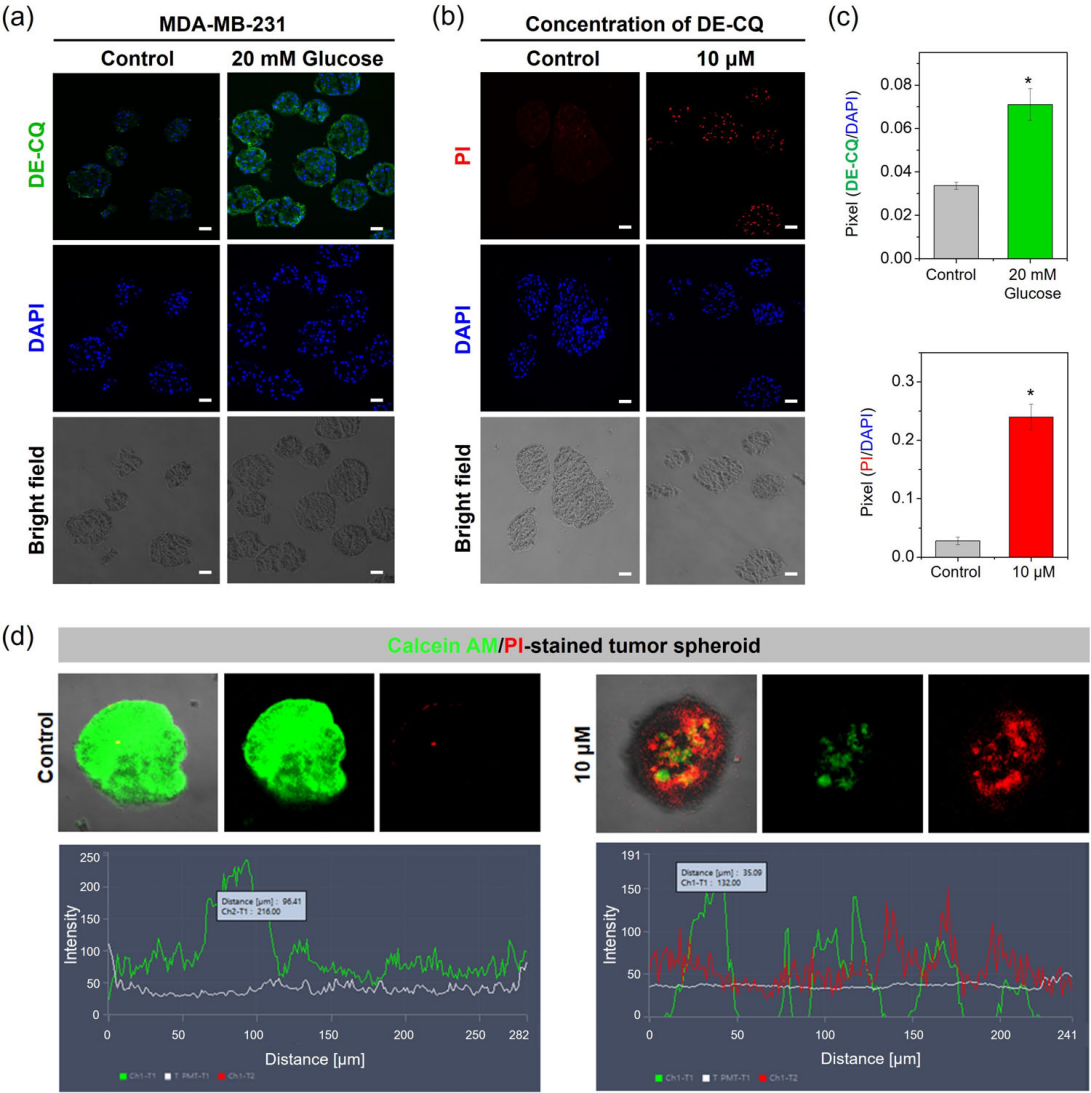
The theranostic effect of DE‐CQ in MDA‐MB‐231 tumor spheroids. (a) Confocal fluorescence imaging of DE‐CQ in tumor spheroids. Cryosectioned tumor spheroids were treated with 20 mM glucose for 45 min, incubated with 10 µM DE‐CQ for 15 min. Bright field indicates a corresponding nonfluorescent phase contrast image. Scale bar: 50 µm. (b) Therapeutic potential of DE‐CQ. Tumor spheroids were incubated with 10 µM DE‐CQ for 12 h and stained with PI for 2 h to visualize dead cells. Scale bar: 50 µm. (c) Quantitative analysis of the fluorescent intensities of DE‐CQ‐treated cells (green) and PI‐stained dead cells (red) in cryosectioned tumor spheroids. (d) Live/dead cell imaging of tumor spheroids incubated with DE‐CQ.

To further evaluate the in vivo antitumor activity of **DE‐CQ**, a xenograft mouse model was established by subcutaneously inoculating MDA‐MB‐231 cells into the left flank of BALB/c nude mice. Following the experimental protocol (Figure 7a), treatment was initiated once the tumors reached a mean volume of approximately 50 mm^3^. Body weight was monitored daily after intraperitoneal (IP) administration of **DE‐CQ** (5 and 10 mg/kg), which showed no significant changes, suggesting negligible systemic toxicity (Figure 7b). Notably, **DE‐CQ** treatment led to a dose‐dependent and significant suppression of tumor growth compared to the vehicle control group (Figure 7c). Upon sacrifice at day 10, the excised tumors from the **DE‐CQ**‐treated mice exhibited a marked reduction in both volume and weight (Figure 7d,e). These findings demonstrate that **DE‐CQ** exerts potent in vivo therapeutic efficacy against aggressive breast cancer tumors.

**FIGURE 7:**
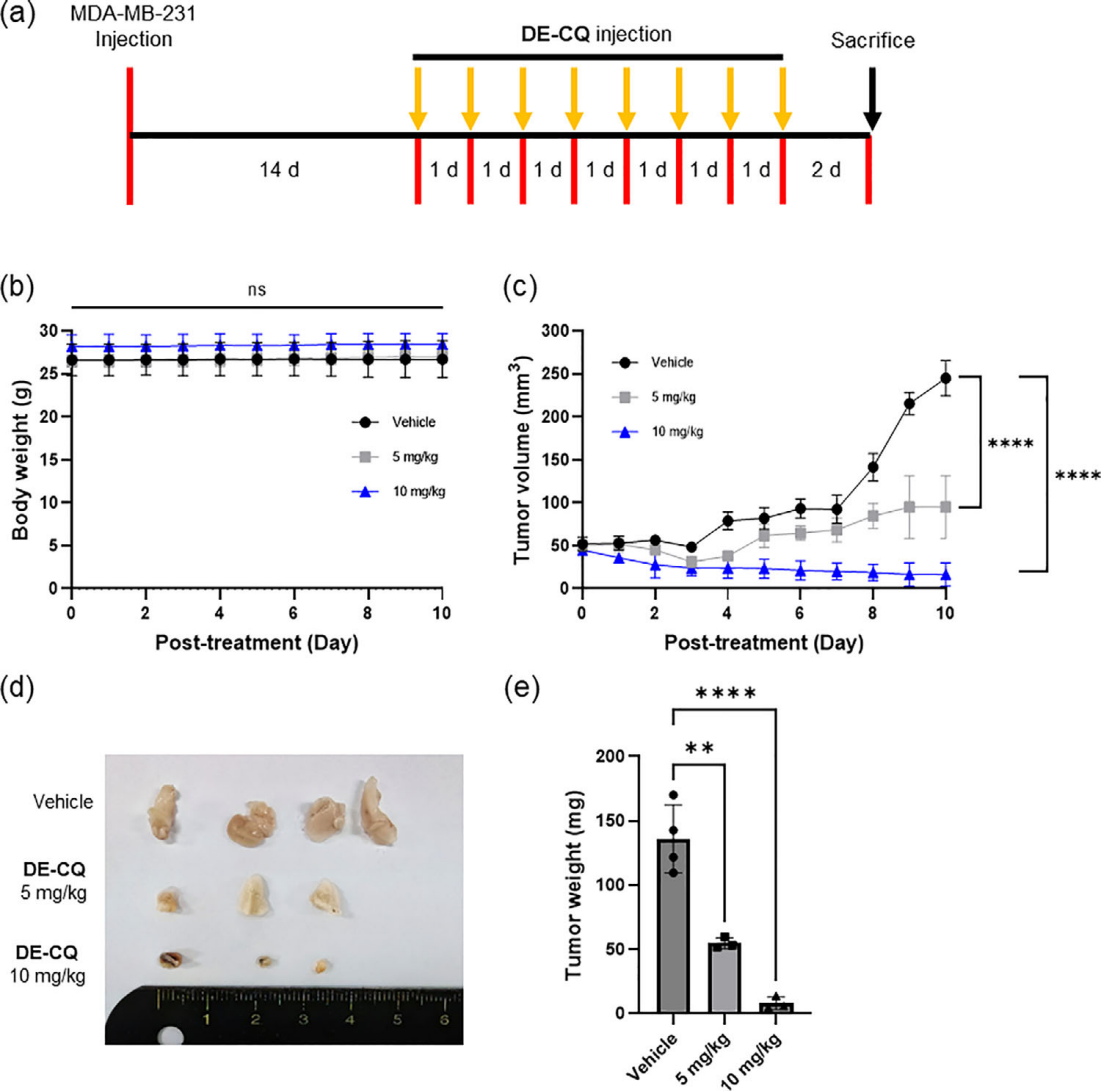
DE‐CQ exhibits antitumor activity in an MDA‐MB‐231 xenograft mouse model. (a) Scheme of the xenograft mouse experiment. (b) Body weight was measured daily during treatment with DE‐CQ (5 and 10 mg/kg). (c) Tumor volume was measured daily during treatment with DE‐CQ (5 and 10 mg/kg). Data were analyzed using two‐way ANOVA followed by Dunnett's multiple comparisons test for comparisons between treatment groups and the vehicle control at each time point. (d) Representative images of excised tumors. (e) Tumor weight was measured 10 days after the first treatment with DE‐CQ (5 and 10 mg/kg). Data were analyzed using one‐way ANOVA followed by Dunnett's multiple comparisons test for comparisons between treatment groups and the vehicle control. Data are presented as mean ± SEM. Vehicle, n = 4; DE‐CQ 5 mg/kg, n = 3; DE‐CQ 10 mg/kg, n = 3. **p < 0.01, ****p < 0.0001 compared to the vehicle control.

The efficacy of **DE‐CQ** as a theranostic agent was further validated through in vivo animal studies, where both imaging and therapeutic evaluations in MDA‐MB‐231 xenograft models yielded highly promising results. In summary, we have successfully developed the NAD(P)H‐activatable probe **DE‐CQ** and comprehensively validated its multifaceted potential as a diagnostic tool for identifying aggressive cancer cells, and as a therapeutic agent with proven cancer‐killing effects. These capabilities were rigorously demonstrated across 2D cell cultures, 3D tumor spheroids, and in vivo mouse models. Consequently, as summarized in Table S1, **DE‐CQ** distinguishes itself from conventional NAD(P)H probes by offering remarkably enhanced diagnostic sensitivity and potent apoptosis‐mediated therapeutic efficacy. Given its dual theranostic modality and high selectivity, the **DE‐CQ** scaffold represents a promising chemical platform for the development of next‐generation theranostic agents for aggressive cancers.

## 3 Conclusion

We have developed **DE‐CQ** as a sophisticated NAD(P)H‐activatable fluorophore that demonstrates dual functionality in both diagnostic cancer imaging and chemotherapy. **DE‐CQ** effectively penetrates cancer cells, providing a robust and quantitative fluorescence response to elevated NAD(P)H levels, a key metabolic signature of upregulated glycolysis. Beyond its sensing capabilities, **DE‐CQ** exerts potent antitumor activity by inducing mitochondria‐ and ER stress‐dependent, caspase‐mediated apoptosis, accompanied by intracellular ROS accumulation and mitochondrial membrane depolarization. **DE‐CQ** also suppressed the migratory and invasive properties of MDA‐MB‐231 cells, further supporting its therapeutic potential against aggressive cancer phenotypes. Notably, **DE‐CQ** exhibited superior cancer‐specific cytotoxicity in MDA‐MB‐231 cells compared to normal cells, highlighting its favorable therapeutic index. The efficacy of **DE‐CQ** was rigorously validated across multiple biological platforms, ranging from 2D cell cultures to 3D tumor spheroids to in vivo xenograft models. Collectively, these findings position **DE‐CQ** as a highly effective platform for the simultaneous diagnosis and treatment of aggressive cancers, offering a promising foundation for the advancement of personalized molecular theranostics.

## Author Contributions

**Yujin Cha**: methodology, data curation, investigation, validation, formal analysis, writing – original draft, writing – review and editing. **Taehoon Oh**: methodology, data curation, investigation, validation, formal analysis, writing – review and editing, writing – original draft. **Hui Joo Kim**: methodology, data curation, investigation, validation, formal analysis, writing – review and editing, writing – original draft. **Minju Kang**: methodology, data curation, investigation, validation, writing – review and editing. **Shin A Yoon**: methodology, data curation, investigation, validation. **Yul Lee**: methodology, data curation, investigation. **Yeon‐Ju Lee**: investigation, methodology, data curation. **Sun Young Park**: methodology, investigation. **Dong‐Sik Shin**: writing – review and editing, funding acquisition, project administration, supervision, methodology, validation, data curation. **Sung‐Kyun Ko**: supervision, funding acquisition, project administration, writing – review and editing, methodology, investigation, validation, formal analysis, data curation, writing – original draft. **Jiyou Han**: conceptualization, methodology, funding acquisition, writing – review and editing, project administration, data curation, investigation, validation, writing – original draft, formal analysis. **Min Hee Lee**: conceptualization, methodology, data curation, investigation, validation, project administration, supervision, funding acquisition, writing – original draft, writing – review and editing, formal analysis.

## Conflicts of Interest

The authors declare no conflicts of interest.

## Supporting information




**Supporting File**: anie74365‐sup‐0001‐SuppMat.docx.

## Data Availability

The data that supports the findings of this study are available in the Supporting Information of this article.
