# Supplementary material for: Theranostic Potential of an NAD(P)H‐Activatable Fluorophore: Assessing Cancer Aggressiveness and Triggering Apoptosis
Source: Angew Chem Int Ed Engl. 2026 Aug 25;65(42):e7968058. doi: 10.1002/anie.7968058 (PMC13653622; doi:10.1002/anie.7968058)
Supplement: Supplementary file 1 [file ANIE-65-e7968058-s001.docx]

Supporting Information

*for*

**Theranostic Potential of an NAD(P)H-Activatable Fluorophore: Assessing Cancer Aggressiveness and Triggering Apoptosis**

Yujin Cha^a,^*^ǂ^*, Taehoon Oh^b,c,^*^ǂ^*, Hui Joo Kim^d,^*^ǂ^*, Minju Kang^d^, Shin A Yoon^e^, Yul Lee^f^, Yeon-Ju Lee^f^, Sun Young Park^a^, Dong-Sik Shin^f,*^, Sung-Kyun Ko^b,g,*^, Jiyou Han^h,*^, and Min Hee Lee^d,*^

*^a^Department of Chemistry, Sookmyung Women’s University, Seoul 04310, Republic of Korea*

*^b^Chemical Biology Research Center, Korea Research Institute of Bioscience and Biotechnology (KRIBB), Cheongju 28116, Republic of Korea
^c^College of Pharmacy, Chungbuk National University, Cheongju 28644, Republic of Korea
^d^Department of Chemistry, Chung-Ang University, Seoul 06974, Republic of Korea*

*^e^Therapeutics & Biotechnology Division, Korea Research Institute of Chemical Technology (KRICT), Daejeon 34114, Korea*

*^f^Department of Chemical and Biological Engineering, Sookmyung Women’s University, Seoul 04310, Republic of Korea*

*^g^KRIBB School of Bioscience, University of Science and Technology (UST), Daejeon 34113, Republic of Korea*

*^h^Department of Biomedical and Chemical Sciences, Laboratory of Stem Cell Research and Biotechnology, Hyupsung University, Hwaseong-si, 18330, Republic of Korea*

*^ǂ^These authors are equally contributed to this work.*

*Corresponding authors: dshin@sookmyung.ac.kr (D.-S. Shin), ksk1230@kribb.re.kr (S.-K. Ko), [hanjiyou12@hanmail.net](mailto:hanjiyou12@hanmail.net) (J. Han), minheelee@cau.ac.kr (M. H. Lee)

**Contents**

1. **Materials and instrumentation**
2. **UV/Vis absorption and fluorescence spectroscopic methods**
3. **Kinetic analysis**
4. **Time-resolved photoluminescence (TR-PL) measurements**
5. **Cell culture and fluorescence microscopic methods**
6. **Cytotoxicity assay**
7. **Tumor spheroid formation and PI staining**
8. **Time-dependent fluorescence imaging and ATP-based viability assay of tumor spheroids**
9. **Gene expression (real-time PCR)**
10. **Intracellular ROS measurement using H_2_DCF-DA**
11. **Measurement of mitochondrial membrane potential using DiOC_6_**
12. **Western blot analysis**
13. **Annexin V staining**
14. **Co-staining of compound and organelle trackers and confocal microscopic methods**
15. **Scratch wound healing assay**
16. **Transwell chamber invasion assay**
17. **Time-lapse cell tracking analysis**
18. **Animal studies**
19. **Xenograft studies in mice**
20. **Statistical analysis**
21. **Synthesis of compounds**
22. **Comparison of DE-CQ with previously reported NAD(P)H-sensitive probes**
23. **^1^H and ^13^C NMR and ESI-MS analysis**
24. **Additional data**
25. **References**

**1. Materials and instrumentation**

All reagents were purchased from Sigma-Aldrich (St. Louis, MO, USA), Alfa (Alfa, Heysham, United Kingdom), or TCI (TCI, Tokyo, Japan) and used without further purification. M-PER buffer (#78501), SuperSignal West Pico Chemiluminescent Substrate (#34080), and SuperSignal West Femto Maximum Sensitivity Substrate (#34095) were purchased from Thermo Fisher Scientific (Waltham, MA, USA). Primary antibodies against Caspase-9 (#9502), Caspase-3 (#9665), and PARP (#9542) were purchased from Cell Signaling Technology (Danvers, MA, USA), while β-actin antibody (sc-47778) was purchased from Santa Cruz Biotechnology (Dallas, TX, USA). All primary antibodies were used at a dilution of 1:1000. Horseradish peroxidase (HRP)-conjugated anti-mouse IgG (#7076) and anti-rabbit IgG (#7074) secondary antibodies were purchased from Cell Signaling Technology (Danvers, MA, USA) and used at a dilution of 1:5000. All solvents were analytical or HPLC grade. HPLC analysis was conducted using Shimadzu HPLC apparatus (Shimadzu LC 6AD; Kyoto, Japan) with a Thermo-Fisher Scientific (Waltham, MA, USA) Acclaim™ 120 C18 (3 μm, 120 Å, 2.1 × 150 mm) column. The flow rate was 0.5 mL/min. For the mobile phase, Buffer A (H_2_O with 0.1% v/v trifluoroacetic acid) and Buffer B (acetonitrile with 0.1% v/v trifluoroacetic acid) were used for the solvent gradient. All fluorescence and UV/Vis absorption spectra were collected using RF-6000 (Shimadzu) and UV-2600 (Shimadzu Corporation) spectrophotometer, respectively. ^1^H and ^13^C nuclear magnetic resonance (NMR) data were obtained using a Bruker instrument (500 MHz, Bruker Corporation, Billerica, MA, USA). High-resolution electrospray ionization mass spectrometry (HR-ESI-MS) data were measured using liquid chromatography mass spectrometry (LC/MS) at the Korea Basic Science Institute (KBSI, Seoul).

**2. UV/Vis absorption and fluorescence spectroscopic methods**

Stock solutions of synthetic compounds were prepared in dimethyl sulfoxide (DMSO). Stock solutions of metal chloride salts and thiols were prepared in deionized water. Stock solutions of tetrabutylammonium (TBA) salt anions were prepared in CH_3_CN. Stock solutions of reactive oxygen species (ROS) were prepared using procedures from the literature.^1^ Stock solutions of glucose, adenosine triphosphate (ATP), adenosine diphosphate (ADP), nicotinamide adenine dinucleotide (NAD^+^), reduced nicotinamide adenine dinucleotide phosphate (NADPH), and reduced nicotinamide adenine dinucleotide (NADH) were prepared in deionized water. All spectra were recorded in a phosphate buffered saline (PBS) solution (pH 7.4, 10 mM) containing 1 mM cetyltrimethylammonium bromide (CTAB) and 1% (v/v) DMSO. Excitations were carried out at 490 nm for **DE-CQ** and 465 nm for **CQ** with excitation slit widths of 5 nm.

**3. Kinetic analysis**

Time-dependent fluorescence measurements were performed to investigate the reduction kinetics of **DE-CQ** and **CQ** by NADH. **DE-CQ** and **CQ** (each 5 μM) were incubated with NADH (50 μM) in PBS (10 mM, pH 7.4) containing 1 mM CTAB and 1% (v/v) DMSO at 37 °C. The fluorescence intensity was monitored for 120 min, with excitation wavelengths of 490 and 465 nm for **DE-CQ** and **CQ**, respectively. The time-dependent fluorescence data were fitted using OriginPro8.5 according to the following equation, where *k* represents the observed rate constant (*k_obs_*).

$$f\left( t \right)=f\left( 0 \right)+e^{-kt}$$

**4. Time-resolved photoluminescence (TR-PL) measurements**

The TR-PL study was carried out using a confocal microscope (MicroTime-200, Picoquant, Germany). The PL lifetime measurements were performed at the Korea Basic Science Institute (KBSI), Korea. A single-mode pulsed diode laser (375 or 470 nm with an average power of ~50 μW operating in 20 MHz repetition rate) with 30 ps pulse width was used as an excitation source. The pulsed laser line was focused on samples through a 40× objective. Emission photons were directed to a dichroic mirror (Z375RDC or 490 DCXR, AHF), a long pass filter (HQ405lp or HQ500lp, AHF), a 150 μm pinhole, a band-pass filter (FEL0550, Thorlabs), and finally collected by an avalanche photodiode detector (PDM series, MPD). A time-correlated single-photon counting system (PicoHarp300, PicoQuant GmbH, Germany) was used to count emission photons using the time-tagged time-resolved (TTTR) data acquisition method. Exponential function fittings for the obtained PL decays were performed using the Symphotime-64 software (Ver. 2.2) by the exponential decay model; $I(t)=\sum A_{i}e^{-t/\tau_{i}}$, where *I(t)* is the time-dependent PL intensity, *A* is the amplitude, and τ is the PL lifetime.

**5. Cell culture and fluorescence microscopic methods**

MDA-MB-231 (human breast cancer), T47D (human breast cancer), DU-145 (human prostate cancer), HuH-7 (human liver cancer), A549 (human lung cancer), HCT-116 (human colon cancer) cell lines were cultured in Roswell Park Memorial Institute (RPMI) 1640 medium supplemented with 10% fetal bovine serum (FBS) and 100 U/mL penicillin-streptomycin. HeLa (human cervical cancer), PANC-1 (human pancreatic cancer), AGS (human gastric cancer), and WI-38 (human fibroblast cell) cell lines were cultured in Dulbecco’s modified Eagle’s medium (DMEM) supplemented with 10% FBS and 100 U/mL penicillin-streptomycin. NIH-3T3 (mouse fibroblast cell) was cultured in DMEM supplemented with 10% bovine calf serum (BCS) and 100 U/mL penicillin-streptomycin. MCF-7 (human breast cancer), LNCaP-LN3 (human prostate cancer), and HepG2 (human liver cancer) cell lines were cultured in Minimum Essential Medium (MEM) supplemented with 10% FBS and 100 U/mL penicillin-streptomycin. All cells were maintained in a humidified atmosphere containing 5% CO_2_ at 37 °C. Two days before the microscopic experiments, the cells were transferred to cover glass-bottomed dish. The cells were seeded at 1.0 × 10^5^ per dish and maintained at 37 °C, 5% CO_2_. After 1 d, 10 µM **DE-CQ** or 10 µM **CQ** (from 1 mM stock in DMSO) were added to the cells. The fluorescent images of the cells were obtained after incubation at 37 °C for 15 min. For glucose/pyruvate stimulation experiments, cells were pretreated with 20 mM glucose or 5 mM pyruvate for 45 min, and then incubated with 10 μM **DE-CQ** or 10 μM **CQ** for 15 min. Cells were purchased from the Korean Cell Line Bank (Seoul, South Korea). For cell culture media, RPMI, DMEM, MEM, FBS, BCS, trypsin 0.25%-EDTA and penicillin-streptomycin were purchased from BIOWEST (Nuaillé, France). The clear and adhesion-typed confocal dishes (diameter = 35 mm) were purchased from SPL (Phocheon-si, Korea). Fluorescence images were obtained using a Carl Zeiss Axio Observer 7 and Zeiss apotome.2 (Oberkochen, Germany).

**6. Cytotoxicity assay**

Cytotoxicity was evaluated using an MTT assay. Thiazolyl blue tetrazolium bromide (MTT) was purchased from Sigma-Aldrich and used without further purification. Cells were seeded in 96-well flat-bottomed plates at 1.0 × 10^4^ cells per well and incubated (37 ℃, 5% CO_2_) overnight. Then, cells were treated with different concentrations of the compound, and incubated (37 ℃, 5% CO_2_) for 24 h. Compound was then removed from the cells and washed with PBS three times. Afterwards, MTT reagent (5 mg/mL) was added to each well and incubated (37 ℃, 5% CO_2_) for 3 h. The MTT reagent was removed from the cells, and DMSO (100 µL/well) was added to dissolve the formazan crystals. UV/Vis absorbance was obtained at 540 nm by using Spectra Max i3x microplate reader (Molecular Devices, San Jose, CA, USA).

**7. Tumor spheroid formation and PI staining**

MDA-MB-231 cells were counted 1 × 10^6^ cells and seeded on ultra-low attached 96-well plates (Thermo Fisher Scientific, Inc.) for tumor spheroid formation, following the manufacturer’s instructions. After seeding for 24 h on dishes, the cells were treated with 10 µM **DE-CQ** for 12 h, then the culture medium was exchanged with fresh culture medium. To examine cytotoxicity, the tumor spheroids were incubated with 1.67 μg/mL propidium iodide (PI, Invitrogen; Thermo Fisher Scientific, Inc.) for 2 h at 37 °C. After PI incubation, the tumor spheroids were collected in a microtube. The tumor spheroids were then incubated in 20% sucrose overnight at 4 ℃ for cryo-protection. The fixed tumor spheroids were embedded in optimum cutting temperature (OCT) compound (Tissue-Tek, Sakura Finetek, Tokyo, Japan) and cut into 6 μm-thick sections using a cryostat (Leica CM1950, Leica Biosystems, Wetzlar, Germany). The cryosectioned spheroids were air-dried and counter-stained with 1 μg/mL 4′,6-diamidino-2-phenylindole (DAPI; Roche, Indianapolis, IN, USA) and mounted for confocal microscopy (LSM 510 Meta, Carl Zeiss).

**8. Time-dependent fluorescence imaging and ATP-based viability assay of tumor spheroids**

MDA-MB-231 cells were counted 1 × 10^6^ cells and seeded on ultra-low attached 96-well plates (Thermo Fisher Scientific, Inc.). Spheroid viability was assessed using the CellTiter-Glo® 3D Cell Viability Assay (Promega Corporation, Madison, WI, USA) according to the manufacturer’s instructions. At each designated time point, an equal volume of CellTiter-Glo® 3D reagent (180 pM) was added to the culture medium for 30 min incubation. Green fluorescence images were acquired using a 488 nm excitation laser and a 493–520 nm emission band-pass filter. Green-positive pixels and integrated fluorescence intensity were quantified under identical image-acquisition and analysis settings for all experimental groups. Then the plate was vigorously agitated on an orbital shaker for 5 min to facilitate disruption and complete lysis of the spheroids. The plate was subsequently incubated at room temperature for 25 min to stabilize the luminescent signal, after which luminescence was measured using a microplate reader.

**9. Gene expression (real-time PCR)**

To examine the potential mode of action of **DE-CQ**, various cancer cells and tumor spheroids of MDA-MB-231 cells were collected after 24 h incubation and irradiation. The total RNA from these cells and tumor spheroids was isolated using 200 μL of the TRIzol Reagent (Invitrogen). Traditional reverse transcription studies (Promega, Madison, WI, USA) and real-time PCR (Agilent Technologies, Santa Clara, CA, USA) were carried out according to the manufacturer’s instructions. Takara Ex Taq DNA polymerase (Takara, Kusatsu, Japan) and SYBR® Green Master Mix (Bio-Rad, Hercules, CA, USA) were utilized for the PCR amplification of different genes using a program of 94 °C for 5 minutes, 35 cycles of 94 °C for 30 seconds, 60 °C for 30 seconds, 72 °C for 30 seconds, and extension at 72 °C for 10 minutes. The results were normalized to the β-actin control. The primers and PCR conditions used are shown in Table S3.

**10. Intracellular ROS measurement using H_2_DCF-DA**

MDA-MB-231 cells were seeded in 6-well plates at a density of 1.5 × 10^5^ cells/well and cultured overnight. For time-dependent analysis, cells were treated with 10 μM DE-CQ for 1, 3, 6, 12, or 24 h. DMSO-treated and unstained cells were included as vehicle and fluorescence-negative controls, respectively. For dose-dependent analysis, cells were treated with **DE-CQ** at the indicated concentrations for 24 h. To assess the effect of antioxidant treatment, cells were co-treated with 20 μM **DE-CQ** and N-acetyl-L-cysteine (NAC; A7250, Sigma-Aldrich, St. Louis, MO, USA) 5 mM for 24 h. 2′,7′-Dichlorodihydrofluorescein diacetate (H_2_DCF-DA; D399, Invitrogen, Carlsbad, CA, USA) was added directly to the culture medium at a final concentration of 10 μM during the final 30 min at 37 ℃ of each treatment. Cells were then washed twice with DPBS, detached using trypsin-EDTA, collected by centrifugation at 1,000 rpm for 3 min, and resuspended in DPBS. DCF fluorescence was measured in the FITC channel using a CytoFLEX flow cytometer (Beckman Coulter, Brea, CA, USA), and the data were analyzed using FlowJo v10 software (BD Biosciences, Franklin Lakes, NJ, USA).

**11. Measurement of mitochondrial membrane potential using DiOC_6_**

MDA-MB-231 cells were seeded in 6-well plates at a density of 1.5 × 10^5^ cells/well and cultured overnight. Cells were treated with DMSO or the indicated concentrations of **DE-CQ** for 24 h. To examine the effect of antioxidant treatment, an additional group was co-treated with 10 μM **DE-CQ** and NAC 5 mM for 24 h. Following treatment, cells were incubated with 3,3′-dihexyloxacarbocyanine iodide (DiOC_6_; D273, Invitrogen, Carlsbad, CA, USA) at a final concentration of 30 nM for 30 min at 37 ℃. Cells were then washed twice with DPBS, detached using trypsin-EDTA, collected by centrifugation at 1,000 rpm for 3 min, and resuspended in DPBS. DiOC_6_ fluorescence was measured in the FITC channel using a CytoFLEX flow cytometer, and the data were analyzed using FlowJo v10 software.

**12. Western blot analysis**

MDA-MB-231 cells were lysed in cold M-PER buffer supplemented with protease inhibitor cocktail and phosphatase inhibitor cocktail 3, followed by centrifugation at 13,500 rpm for 20 min at 4°C. Protein concentrations were determined using a Pierce BCA Protein Assay Kit (Thermo Fisher Scientific, Waltham, MA, USA). Equal amounts of protein (30 μg per lane) were separated on 10% SDS-PAGE gels and transferred onto nitrocellulose membranes (0.45 μm; 10600002, Cytiva, Marlborough, MA, USA). The membranes were blocked with 5% non-fat dry milk in Tris-buffered saline containing 0.1% Tween 20 (TBST) for 1 h at room temperature and incubated with the indicated primary antibodies at 4°C overnight. After washing with TBST, the membranes were incubated with the corresponding HRP-conjugated secondary antibodies for 2 h at room temperature. Protein bands were visualized using enhanced chemiluminescence substrates.

**13. Annexin V staining**

MDA-MB-231 cells were seeded to 6-well plates (1.5 × 10^5^ cells/well) and treated with 0, 10, 25 and 50 µM DE-CQ for 24 h. After treatment, cells were washed with PBS, detached using trypsin-EDTA (0.5 mL, 0.05% trypsin, 0.02% EDTA, Sigma-Aldrich, Saint Louis, MO, USA) for 3 min at 37 ℃, and collected by centrifugation at 1,000 rpm for 3 min. The cells were resuspended in a binding buffer (500 μL, 10 mM HEPES/NaOH, pH 7.5 containing 140 mM NaCl and 2.5 mM CaCl_2_) and stained with annexin V-FITC (0.5 μg/mL) for 15 min at room temperature. The stained cells were determined using the BD FACSCalibur™ flow cytometer (BD Biosciences, Franklin Lake, NJ, USA) and the data were analyzed using FlowJo v10 (BD Biosciences, Franklin Lake, NJ, USA).

**14. Co-staining of compound and organelle trackers and confocal microscopic methods**

Organelle tracking dyes were purchased from Invitrogen. In experiments using **DE-CQ**, MDA-MB-231 cells were pretreated with Mito-Green (0.1 μM), Lyso-Green (0.05 μM), or ER-Green (0.1 μM), in RPMI media for 15 min at 37 °C. The cells were then incubated with **DE-CQ** (10 μM) for 25 min at 37 °C. The excitation wavelength and filter set were 488 nm with a 644–700 nm BP for **DE-CQ** and 488 nm with a 493–520 nm BP for organelle trackers, respectively. Fluorescence images were obtained using Zeiss LSM-700 with a 40× objective (1x and 1.5x zoom) (Carl Zeiss). Image analysis was performed using ZEN software and PCC values were obtained by using AutoQuant X3 software.

**15. Scratch wound healing assay**

MDA-MB-231 cells were seeded in 24-well plates at a density of 6.0 × 10^4^ cells/well and cultured for 12 h. A linear scratch was generated across the center of each well using a sterile 200 μL pipette tip. Cells were treated with mitomycin C (10 μg/mL) for 3 h to minimize the contribution of cell proliferation and subsequently treated with **DE-CQ** at the indicated concentrations for 24 h. A mitomycin C-treated group was included as a negative control. Following treatment, the cells were fixed with 4% paraformaldehyde for 12 min, stained with 0.2% crystal violet, and imaged using a microscope (ZEISS, Germany). The residual wound width at 24 h was measured using ImageJ software (version 1.53k; Wayne Rasband, National Institutes of Health, USA) and normalized to the mitomycin C-treated control.

**16. Transwell chamber invasion assay**

Cell invasion was assessed using 6.5-mm Transwell inserts with an 8.0 μm pore size (3422; Corning, USA). The upper surface of each insert was coated with Matrigel, and MDA-MB-231 cells (6.0 × 10^4^ cells/well) were seeded into the upper chamber in 200 μL of serum-free medium containing DMSO or the indicated concentrations of **DE-CQ**. The lower chamber was filled with serum-containing DMEM as a chemoattractant. Serum-free medium was used as a negative control. After incubation for 24 h, non-invasive cells remaining on the upper surface of the membrane were removed. Cells that had invaded through the membrane were fixed with 4% paraformaldehyde for 12 min, stained with 0.2% crystal violet, and imaged using a microscope (ZEISS, Germany). The stained area was quantified using ImageJ software, and relative invasion was calculated by normalization to the DMSO-treated control.

**17. Time-lapse cell tracking analysis**

MDA-MB-231 cells were seeded at a density of 4.0 × 10^4^ cells in 100 μL of DMEM supplemented with 2% Matrigel in μ-Slide I chambers (80106; Ibidi, Germany) and allowed to attach for 3 h. Subsequently, 900 μL of DMEM containing 5% FBS was added, and the cells were cultured overnight. Cells were then treated with DMSO or the indicated concentrations of **DE-CQ** and immediately transferred to a HoloMonitor M4 time-lapse cytometer (Phase Holographic Imaging, Sweden) maintained at 37 ℃. Images were acquired at 15-min intervals for 12 h, and individual cells were tracked using HoloStudio M4 software. Cells that moved outside the imaging field during acquisition were excluded from the analysis. Cell trajectories and cumulative motility were determined over the 12-h observation period, and total motility at 12 h was quantified from 32 individually tracked cells per group.

**18. Animal studies**

All experiments were approved by the KRIBB Institutional Animal Care and Use Committee (KRIBB-AEC-25412). Five-week-old male BALB/c nude mice were purchased from DBL (Seoul, Korea). The mice were acclimated to the animal facility for 1 week. The mice were maintained under a 12:12 h light/dark cycle in a temperature-controlled environment with free access to food and water.

**19. Xenograft studies in mice**

For the xenograft studies, 3x10^6^ MDA-MB-231 cells were subcutaneously injected into the left flank of mice. When the tumor volume reached approximately 50 mm^3^, the mice were randomly assigned to three groups. Each mouse received intraperitoneal (IP) injections (100 μL per mouse) of vehicle (PBS:ethanol:Tween 80, 8:1:1, containing 0.1% DMSO), prepared by mixing the organic components prior to dilution with PBS, or DE-CQ (5 and 10 mg/kg) daily for a total of eight administrations, according to the experimental scheme in Fig. 7a. The tumor size was measured daily, and tumor volume was calculated using the following equation:

Tumor volume = (length x width^2^)/2

The body weight was measured daily. At 10 days after the first administration, the mice were euthanized, and tumors were excised and weighed.

**20. Statistical analysis**

For all quantitative data, where statistical analysis was performed, statistically meaningful sample sizes were chosen to allow significant differences between controls and treatments with or without **DE-CQ** or **CQ**. All *in vitro* studies were conducted in triplicate over three independent experiments. The results were presented along with the standard error of the mean. Raw data was normalized using Excel, prior to further analysis. SAS software (version 8.2, Cary, NC, USA) with one-way and two-way analysis of variance (ANOVA) was applied to determine statistical significance. When the ANOVA results indicated a significant difference, a two-tailed Student’s *t*-test and a *post hoc* Bonferroni test were performed using SAS software or the Origin software package (version 9.4, Northampton, MA, USA). Significant P-values (**P* < 0.05, ***P* < 0.01, and ****P* < 0.001) are reported using asterisks or different letters in the figures and captions.

**21. Synthesis of compounds**

**Synthesis of DE-CQ**: A mixture of **2** (100 mg, 0.19 mmol) and methyl trifluoromethanesulfonate (MeOTf) (0.22 mL, 1.94 mmol) in CHCl_3_ (10 mL) was stirred for 1 h at room temperature. The solvent was then removed *in vacuo*. The crude product was purified on silica gel using CH_2_Cl_2_/MeOH (v/v, 50:1) as the eluent to yield **DE-CQ** as a brown solid (55 mg, 53%). HR-ESI-MS m/z [M]^+^ calc. 527.2540, [M]^+^ obs. 527.2571. ^1^H NMR (DMSO-*d_6_*, 500 MHz): *δ* 10.05 (s, 1H), 9.62 (s, 1H), 8.52 (d, *J* = 8.9 Hz, 1H), 8.40 (d, *J* = 7.6 Hz, 1H), 8.28 (t, *J* = 8.6 Hz, 1H), 8.07 (m, 2H), 7.87 (t, *J* = 4.6 Hz, 2H), 7.62 (d, *J* = 16.2 Hz, 1H), 6.74 (d, *J* = 11.5 Hz, 1H), 6.35 (d, *J* = 2.5 Hz, 1H), 4.67 (s, 3H), 4.28 (m, 2H), 4.16 (m, 2H), 3.46 (m, 4H), 1.30 (t, *J* = 7.0 Hz, 3H), 1.23 (t, *J* = 7.0 Hz, 3H), 1.14 (t, *J* = 7.0 Hz, 6H) ppm. ^13^C NMR (DMSO-*d_6_*, 125 MHz): *δ* 165.3, 164.4, 161.3, 154.0, 150.6, 150.0, 143.4, 140.5, 137.4, 135.4, 130.5, 130.2, 129.0, 128.7, 127.4, 125.8, 122.0, 119.3, 108.9, 107.2, 106.2, 98.6, 96.7, 60.4, 59.6, 45.6, 44.0, 14.3, 14.1, 12.3 ppm.

**Synthesis of CQ:** A mixture of **1** (200 mg, 0.54 mmol) and methyl trifluoromethanesulfonate (MeOTf) (0.3 mL, 2.65 mmol) in CHCl_3_ (10 mL) was stirred for 1 h at room temperature. The solvent was then removed *in vacuo*. The crude product was purified on silica gel using CH_2_Cl_2_/MeOH (v/v, 20:1) as the eluent to yield **CQ** as a red solid (81 mg, 39%). HR-ESI-MS m/z [M]^+^ calc. 385.1911, [M]^+^ obs. 385.1923. ^1^H NMR (DMSO-*d_6_*, 500 MHz): *δ* 10.06 (s, 1H), 9.48 (s, 1H), 8.53 (d, *J* = 8.9 Hz, 1H), 8.45 (d, *J* = 8.2 Hz, 1H), 8.29 (t, *J* = 8.6 Hz, 1H), 8.09 (m, 2H), 7.96 (d, *J* = 9.2 Hz, 1H), 7.83 (d, *J* = 16.2 Hz, 1H), 6.78 (d, *J* = 11.5 Hz, 1H), 6.61 (d, *J* = 2.5 Hz, 1H), 6.43 (s, 1H), 4.69 (s, 3H), 3.48 (m, 4H), 1.15 (t, *J* = 7.0 Hz, 6H) ppm. ^13^C NMR (DMSO-*d_6_*, 125 MHz): *δ* 160.4, 155.7, 150.1, 149.1, 148.3, 143.1, 136.9, 134.9, 130.0, 129.9, 129.7, 129.5, 128.4, 126.0, 125.6, 118.7, 108.1, 105.9, 102.5, 96.7, 45.1, 43.4, 11.7 ppm.

**Synthesis of 1:** A mixture of 7-diethylamino-4-methylcoumarin (500 mg, 2.16 mmol) and potassium *tert*-butoxide (*t*-BuOK) (340 mg, 2.16 mmol) in DMSO (10 mL) was stirred for 1 h at room temperature. Subsequently, 3-quinolinecarboxaldehyde (240 mg, 2.16 mmol) was added to the mixture. The resulting mixture was stirred at room temperature for 5 h. After AcOH (0.2 mL) and water (50 mL) were added, a yellow precipitate was formed, washed with water, and dried *in vacuo*. The yellow solid was dissolved in a small amount of CH_2_Cl_2_ and purified on silica gel using EA/Hexanes (v/v, 1:3) as the eluent to yield **1** as a yellow solid (60 mg, 37%). HR-ESI-MS m/z [M]^+^ calc. 370.1681, [M+H]^+^ obs. 371.1759. ^1^H NMR (CDCl_3_, 500 MHz): *δ* 9.20 (s, 1H), 8.28 (s, 1H), 8.17 (d, *J* = 8.7 Hz, 1H), 7.89 (d, *J* = 8.3 Hz, 1H), 7.77 (t, *J* = 7.5 Hz, 1H), 7.62 (t, *J* = 7.5 Hz, 2H), 7.54 (d, *J* = 16.2 Hz, 1H), 7.43 (d, *J* = 17.6 Hz, 1H), 6.65 (d, *J* = 8.0 Hz, 1H), 6.57 (s, 1H), 6.31 (s, 1H), 3.45 (m, 4H), 1.23 (t, *J* = 7.0 Hz, 6H) ppm. ^13^C NMR (CDCl_3_, 125 MHz): *δ* 162.4, 156.6, 150.8, 149.8, 148.6, 147.6, 134.8, 133.1, 130.4, 129.1, 129.0, 128.2, 127.9, 127.6, 125.3, 123.7, 108.6, 107.5, 104.2, 98.1, 44.8, 12.5 ppm.

**Synthesis of 2:** A mixture of **3** (1 g, 2.68 mmol) and potassium *tert*-butoxide (*t-*BuOK) (300 mg, 2.67 mmol) in DMSO (25 mL) was stirred for 1 h at room temperature. Subsequently, 3-quinolinecarboxaldehyde (500 mg, 3.18 mmol) was added to the mixture. The resulting mixture was stirred at room temperature for 2 h. After AcOH (1.3 mL) and water (100 mL) were added, an orange precipitate was formed, washed with water, and then dried *in vacuo*. The orange solid was dissolved in a small amount of CH_2_Cl_2_ and purified on silica gel using EA/Hexanes (v/v, 1:3) as the eluent to yield **2** as a red solid (90 mg, 13%). HR-ESI-MS m/z [M]^+^ calc. 512.2311, [M+H]^+^ obs. 513.2393, [M+Na]^+^ obs. 535.2205. ^1^H NMR (CDCl_3_, 500 MHz): *δ* 9.16 (s, 1H), 8.24 (s, 1H), 8.11 (d, *J* = 8.4 Hz, 1H), 7.96 (s, 1H), 7.85 (d, *J* = 7.9 Hz, 1H), 7.72 (t, *J* = 8.3 Hz, 1H), 7.58 (t, *J* = 7.9 Hz, 1H), 7.49 (m, 3H), 6.55 (d, *J* = 11.5 Hz, 1H), 6.36 (d, *J* = 2.5 Hz, 1H), 4.39 (m, 2H), 4.25 (m, 2H), 3.39 (m, 4H), 1.40 (t, *J* = 7.1 Hz, 3H), 1.32 (t, *J = 7.1 Hz*, 3H), 1.20 (t, *J = 7.1 Hz*, 6H) ppm. ^13^C NMR (CDCl_3_, 125 MHz): *δ* 166.9, 165.5, 162.6, 154.6, 150.4, 149.0, 147.6, 141.0, 134.2, 131.9, 130.0, 129.3, 129.1, 128.2, 127.9, 127.4, 124.7, 123.8, 108.5, 108.4, 107.6, 98.7, 97.8, 60.8, 60.0, 44.6, 14.4, 14.3, 12.6 ppm.

**Synthesis of 3**: A mixture of diethyl malonate (970 mg, 6.06 mmol) and potassium *tert*-butoxide (*t*-BuOK) (680 mg, 6.06 mmol) in DMSO (8 mL) was stirred for 1 h at room temperature. **4**^2^ (1 g, 4.04 mmol) and silver nitrate (AgNO_3_) (860 mg, 10.10 mmol) were then added to the mixture. The resulting mixture was stirred for 6 h in the dark at room temperature. After EA (200 mL) and water (200 mL) were added to the reaction mixture, the organic layer was collected and dried with anhydrous Na_2_SO_4_. After the removal of the solvents, the crude product was purified over silica gel using EA/Hexanes (v/v, 1:5) as the eluent to yield **3** as a yellow solid (527 mg, 35%). HR-ESI-MS m/z [M]^+^ calc. 373.1889, [M+H]^+^ obs. 374.1978, [M+Na]^+^ obs. 396.1787. ^1^H NMR (CDCl_3_, 500 MHz): *δ* 7.44 (s, 1H), 7.25 (d, *J* = 9.0 Hz, 1H), 6.50 (d, *J* = 9.0 Hz, 1H), 6.32 (s, 1H), 4.36 (m, 2H), 4.21 (m, 2H), 3.37 (m, 4H), 2.27 (s, 1H), 1.37 (t, *J* = 7.2 Hz, 3H), 1.29 (t, *J* = 7.1 Hz, 3H), 1.18 (t, *J* = 7.1 Hz, 6H) ppm. ^13^C NMR (CDCl_3_, 125 MHz): *δ* 167.0, 165.6, 162.8, 154.3, 150.2, 143.8, 125.5, 125.0, 111.1, 110.4, 108.4, 97.4, 60.7, 59.9, 44.6, 18.6, 14.4, 14.3, 12.5 ppm.

**22. Comparison of DE-CQ with previously reported NAD(P)H-sensitive probes**

**Table S1.** Comparison of **DE-CQ** with previously reported NAD(P)H-responsive fluorescent probes.

| Compounds | Ex/Em (nm)  (emission range) | LOD for NADH  (incubation time) | Sensing mechanism  (response manner) | Biological applications | Therapeutic efficacy | Ref. |
| --- | --- | --- | --- | --- | --- | --- |
|  | **Testing** **conditions (buffer pH, solvent system, temp.)** | | |  |  |  |
|   1 | 570/615  (580-700) | 1.2 μM  (3 h) | pyridinium reduction  (fluorescence Off-On) | 2D cell cultures | NO | [3]  (previous work in our group) |
|  | PIPES (pH 7.4, 25 mM, 1 mM CTAB, 2% DMSO) at 37 °C | | |  |  |  |
|   1 | 590/640  (600-750) | 0.10 μM  (80 min) | quinolinium reduction  (fluorescence Off-On) | 2D cell cultures,  3D spheroids | NO | [4]  (previous work in our group) |
|  | PBS (pH 7.4, 10 mM, 1 mM CTAB, 1% DMSO) at 37 °C | | |  |  |  |
|   UQ-Rh | 488/520  (500-600) | 0.5 mM  (10 min) | quinone reduction  in presence of iridium complex  (fluorescence quenching) | 2D cell cultures | NO | [5] |
|  | PBS (pH 7.4, 100 mM) at room temp. | | |  |  |  |
|   RA-Resa | 480/575  (590-750) | 0.41 μM  (20 min) | resazurin derivative reduction, binding of boronic acid/NADH  (fluorescence increase) | 2D cell cultures | NO | [6] |
|  | PBS (pH 7.4, 10 mM) at 37 °C | | |  |  |  |
|   1 | 537/561  (550-700) | 0.1 μM (5 min) | quinolinium reduction  (fluorescence Off-On) | 2D cell cultures  3D spheroids | NO | [7] |
|  | PIPES (pH 7.0, 25 mM, 101 mM NaCl) at 25 °C | | |  |  |  |
|   NAFP4 | 520/584  (530-675) | 3.66 nM  (1 min) | quinolinium reduction  (fluorescence Off-On) | 2D cell cultures | NO | [8] |
|  | PBS (pH 7.4, 10 mM) at room temp. | | |  |  |  |
|   Rh-DD-Q | 760/790  (760-880) | 0.74 μM  (80 min) | quinolinium reduction  (fluorescence Off-On, photoacoustic imaging) | 2D cell cultures,  in vivo | NO | [9] |
|  | PBS (pH 7.4, 10 mM, 0.2% DMSO) at room temp. | | |  |  |  |
|   CQ | 465/575  (500-720) | 0.019 μM  (80 min) | quinolinium reduction  (fluorescence Off-On) | 2D cell cultures | NO | this work |
|  | PBS (pH 7.4, 10 mM, 1 mM CTAB, 1% DMSO) at 37 °C | | |  |  |  |
|   DE-CQ | 490/640  (560-800) | 0.038 μM  (80 min) | quinolinium reduction  (fluorescence Off-On) | 2D cell cultures,  3D spheroids,  in vivo | **Yes** | **this work** |
|  | PBS (pH 7.4, 10 mM, 1 mM CTAB, 1% DMSO) at 37 °C | | |  |  |  |

**23. ^1^H and ^13^C NMR and HR-ESI-MS Analysis**

**Figure S1.** ^1^H NMR spectrum of **DE-CQ** in DMSO-*d_6_*.

**Figure S2.** ^13^C NMR spectrum of **DE-CQ** in DMSO-*d_6_*.

**Figure S3.** HR-ESI-MS spectrum of **DE-CQ**.

**Figure S4.** ^1^H NMR spectrum of **CQ** in DMSO-*d_6_*.

**Figure S5.** ^13^C NMR spectrum of **CQ** in DMSO-*d_6_*.

**Figure S6.** HR-ESI-MS spectrum of **CQ**.

**Figure S7.** ^1^H NMR spectrum of **1** in CDCl_3_.

**Figure S8.** ^13^C NMR spectrum of **1** in CDCl_3_.

**Figure S9.** HR-ESI-MS spectrum of **1**.

**Figure S10.** ^1^H NMR spectrum of **2** in CDCl_3_.

**Figure S11.** ^13^C NMR spectrum of **2** in CDCl_3_.

**Figure S12.** HR-ESI-MS spectrum of **2**.

**Figure S13.** ^1^H NMR spectrum of **3** in CDCl_3_.

**Figure S14.** ^13^C NMR spectrum of **3** in CDCl_3_.

**Figure S15.** HR-ESI-MS spectrum of **3**.

**24. Additional data**

**Figure S16.** Time-dependent fluorescence spectra of (a) **DE-CQ** and (b) **CQ** (5 μM each) in response to the addition of NADH (50 μM) over 120 min in PBS (10 mM, pH 7.4) containing 1 mM CTAB and 1% (v/v) DMSO at 37 °C (λ_ex_ = 490 nm for **DE-CQ**; λ_ex_ = 465 nm for **CQ**).

**Figure S17.** Time-resolved fluorescence decay curves of (a) **DE-CQ** and (b) **CQ** (5 μM each) before and after reaction with NADH (50 μM) for 80 min at 37 °C in PBS (10 mM, pH 7.4) containing 1 mM CTAB and 1% (v/v) DMSO (λ_ex_ = 470 nm).

**Table S2.** Photophysical properties of **DE-CQ** and **CQ** in the absence and presence of NADH.

| Entry | Ф*_f_* ^a^ | *τ* (ns) ^b^ | *k_r_* (s^-1^) ^c^ | *k_nr_* (s^-1^) ^d^ |
| --- | --- | --- | --- | --- |
| **DE-CQ** | 1.05 x 10^-5^ | 1.20 | 8.75 x 10^3^ | 8.33 x 10^8^ |
| **DE-CQ** + NADH | 2.84 x 10^-3^ | 0.65 | 4.37 x 10^6^ | 1.53 x 10^9^ |
| **CQ** | 4.64 x 10^-4^ | 1.36 | 3.41 x 10^5^ | 7.35 x 10^8^ |
| **CQ** + NADH | 3.71 x 10^-2^ | 1.27 | 2.93 x 10^7^ | 7.61 x 10^8^ |

*^a^* Fluorescence quantum yields (Φ*_f_*) were determined in PBS (10 mM, pH 7.4, 1 mM CTAB, 1% v/v DMSO) using 4-amino-*N*-butyl-1,8-naphthalimide (Φ*_f_* = 0.64 in ethanol) as the standard material.

*^b^* Time-resolved fluorescence lifetimes (*τ*) were measured under the same conditions.

*^c,d^* Radiative (*k_r_*) and non-radiative (*k_nr_*) decay rate constants were calculated using *k_r_* = Φ*_f_*/*τ* and *k_nr_* = (1 − Φ*_f_*)/*τ*, respectively.

**Figure S18.** Time-dependent fluorescence intensity changes of (a) **DE-CQ** and (b) **CQ** (5 μM each) at 640 nm and 575 nm, respectively, upon addition of NAD⁺ (50 μM) after pre-incubation with NADH (50 μM) for 80 min at 37 °C in PBS (10 mM, pH 7.4) containing 1 mM CTAB and 1% (v/v) DMSO. Excitation wavelengths were applied at 490 nm and 465 nm for **DE-CQ** and **CQ**, respectively.

**Figure S19.** Fluorescence intensity (FI) of (a) **DE-CQ** and (b) **CQ** (both 5 µM) at an NADH concentration range of 0–100 µM. All data were obtained after 80 min incubation at 37 °C in PBS solution (pH 7.4, 10 mM) containing 1 mM CTAB and 1% (v/v) DMSO. Excitations were applied at 490 nm and 465 nm for **DE-CQ** and **CQ**, respectively.

**Figure S20.** Frontier molecular orbitals, energy gaps (ΔE), and oscillator strengths (*f*) of (a) **DE-CQ** / **DE-CQ**+NADH and (b) **CQ** / **CQ**+NADH, calculated by TD-DFT using the B3LYP functional with 6-31G(d) basis set in Gaussian 09.

**Figure S21.** ESI-MS data of **DE-CQ** and **CQ** after incubation with NADH.

**Figure S22.** Time-dependent fluorescence imaging of MDA-MB-231 cells treated with **DE-CQ** or **CQ** (10 μM, respectively) for 0–30 min. All fluorescence images were collected by the excitation and emission filter sets; λ_em_: 575–640 nm and λ_ex_: 540–552 nm (for **DE-CQ**) or λ_em_: 500–550 nm and λ_ex_: 450–490 nm (for **CQ**), respectively. Bright field indicates a nonfluorescent phase contrast image. Scale bar: 50 μm.

**Figure S23.** Dose-dependent fluorescence imaging of MDA-MB-231 cells treated with **DE-CQ** or **CQ** (0–30 μM, respectively) for 15 min. All fluorescence images were collected by the excitation and emission filter sets; λ_em_: 575–640 nm and λ_ex_: 540–552 nm (for **DE-CQ**) or λ_em_: 500–550 nm and λ_ex_: 450–490 nm (for **CQ**), respectively. Bright field indicates nonfluorescent phase contrast images. Scale bar: 50 μm.

Figure S24. Fluorescence microscopy imaging of various cancer cells before and after glucose stimulation using DE-CQ. Cells were treated with 20 mM glucose for 45 min, and then incubated with 10 μM DE-CQ for 15 min. All fluorescence images were collected with the excitation and emission filter set as λ_ex_: 540–552 nm and λ_em_: 575–640 nm. The bottom image is an overlay of the fluorescent and nonfluorescent phase contrast images. Scale bar: 50 μm.

**Figure S25.** Fluorescence imaging of various cancer cells using **CQ** before and after stimulation with glucose. Various cell lines of (a) breast, (b) cervical, (c) pancreatic, (d) lung, (e) prostate, (f) liver, (g) digestive cancers were used. Cells were treated with 20 mM glucose for 45 min, and then incubated with 10 μM **CQ** for 15 min. All fluorescence images were collected by the excitation and emission filter set λ_em_: 500–550 nm and λ_ex_: 450–490 nm. Bright field indicates nonfluorescent phase contrast images. Scale bar: 50 μm. ****P* < 0.001, n.s.: not significant.

**Figure S26.** Cytotoxicity of **DE-CQ** and **CQ** measured by MTT assay. Normal breast cells MCF10A and breast cancer MDA-MB-231 cells were incubated with various concentrations of **DE-CQ** or **CQ** for 24 h or 48 h.

**Table S3.** Gene primer list used for real-time PCR.

| Name of gene | Forward primer sequence | Reward primer sequence | Annealing Tm (°C)  product size (bp) |
| --- | --- | --- | --- |
| **FAS** | TCTGGTTCTTACGTCTGTTGC | CTGTGCAGTCCCTAGCTTTCC | 60 (130) |
| **Caspase-3** | TGGTGTTGATGATGACATGGCGTG | AGCATGGCACAAAGCGACTGGA | 60 (130) |
| **Caspase-9** | TCGAGGACATCCAGCGGGCA | GCCAGCATGTCCTGGCCTGT | 60 (130) |
| **Bax** | TGGCAGCTGACATGTTTTCTGAC | TCACCCAACCACCCTGGTCTT | 60 (195) |
| **PERK** | GTCCCAAGGCTTTGGAATCTGTC | CCTACCAAGACAGGAGTTCTGG | 60 (143) |
| **CHOP** | GGTATGAGGACCTGCAAGAGGT | CTTGTGACCTCTGCTGGTTCTG | 60 (130) |
| **JNK** | CTTGTGACCTCTGCTGGTTCTG | CTTGTGACCTCTGCTGGTTCTG | 60 (146) |
| **Bim** | GTATTTTTGGTAAATAATGGGGTTG | CAAARAAARCAAAAACRCCCAACA | 60 (139) |
| **Nanog** | CTCCAACATCCTGAACCTCAGC | CGTCACACCATTGCTATTCTTCG | 60 (125) |
| **CD133** | CACTACCAAGGACAAGGCGTTC | CAACGCCTCTTTGGTCTCCTTG | 60 (120) |
| **Snail1** | TGCCCTCAAGATGCACATCCGA | GGGACAGGAGAAGGGCTTCTC | 60 (120) |
| **E-cadherin** | GCCTCCTGAAAAGAGAGTGGAAG | TGGCAGTGTCTCTCCAAATCCG | 60 (130) |
| **MMP9** | GCCACTACTGTGCCTTTGAGTC | CCCTCAGAGAATCGCCAGTACT | 60 (130) |
| **β-actin** | CACCATTGGCAATGAGCGGTTC | AGGTCTTTGCGGATGTCCACGT | 60 (130) |

**Figure S27.** (a) Flow cytometric histograms of H_2_DCF-DA fluorescence in MDA-MB-231 cells treated with 10 μM **DE-CQ** for the indicated times. Unstained and DMSO-treated cells were included as fluorescence-negative and vehicle controls, respectively. (b) Flow cytometric histograms of H_2_DCF-DA fluorescence in cells treated with the indicated concentrations of **DE-CQ** for 24 h. NAC (5 mM) was co-treated with 20 μM **DE-CQ** to assess the effect of antioxidant treatment. (c) Flow cytometric histograms of DiOC_6_ fluorescence in cells treated with the indicated concentrations of **DE-CQ** for 24 h. NAC (5 mM) was co-treated with 10 μM **DE-CQ** to examine its effect on **DE-CQ**-induced changes in mitochondrial membrane potential.

**Figure S28.** Stemness and EMT related gene expression. Various cancer cells were treated with 10 μM **DE-CQ** for 24 h and collected for qPCR analysis. **P* < 0.05.

**Figure S29. DE-CQ** suppresses the migration and invasion of MDA-MB-231 cells. (a) Representative images of scratch wounds in MDA-MB-231 cells treated with DMSO or the indicated concentrations of **DE-CQ** for 24 h and the corresponding quantification of relative wound closure. Mitomycin C was included as a negative control. (b) Representative images of crystal violet-stained MDA-MB-231 cells that invaded through Matrigel-coated Transwell membranes following treatment with DMSO or the indicated concentrations of **DE-CQ** for 24 h and the corresponding quantification of relative cell invasion. Serum-free medium served as a negative control. (c) Trajectories of individually tracked MDA-MB-231 cells treated with DMSO or the indicated concentrations of **DE-CQ** during 12 h of time-lapse imaging. (d) Cumulative cell motility over the 12-h imaging period. (e) Quantification of total cell motility at 12 h. Data in (a) and (b) are presented as the mean ± SD from three independent experiments (n = 3), whereas data in (e) are presented as the mean ± SD (n = 32 cells per group). Statistical significance was determined by one-way ANOVA followed by Dunnett’s multiple-comparisons test. **P* < 0.05, ***P* < 0.01, ****P* < 0.001 and *****P* < 0.0001 versus the DMSO-treated control.

**Figure S30.** Time-dependent fluorescence response and ATP-based viability of MDA-MB-231 tumor spheroids following **DE-CQ** treatment. (a) Confocal fluorescence images of control and **DE-CQ**-treated tumor spheroids, approximately 500 μm diameter at 0, 12, 24 or 48 h. Tumor spheroids were treated with 20 mM glucose for 45 min, and then incubated with 10 μM **DE-CQ** for 15 min. Scale bar: 50 μm. (b) ATP-based viability of control and **DE-CQ**-treated tumor spheroids at the indicated time points, determined using the CellTiter-Glo^®^ 3D Assay (180 pM for 30 min incubation).

**25. References**

1. S. Y. Park, E.-Y. Jo, A. Lee, N. Y. Kim, J. Kim, C. Kang, M. H. Lee, *Sens. Actuators B: Chem.* **2025**, *426*, 137068. DOI: 10.1016/j.snb.2024.137068
2. A. Gandioso, S. Contreras, I. Melnyk, J. Oliva, S. Nonell, D. Velasco, J. García-Amorós, V. Marchán, *J. Org. Chem*. **2017**, *82*, 5398-5408. DOI: 10.1021/acs.joc.7b00788
3. J. H. Joo, D. Youn, S. Y. Park, D. -S. Shin, M. H. Lee, *Dyes Pigm.* **2019**, *170*, 107561. DOI: 10.1016/j.dyepig.2019.107561
4. J. H. Joo, M. Won, S. Y. Park, K. Park, D. -S. Shin, J. S. Kim, M. H. Lee, *Sens. Actuators B: Chem.* **2020**, *320*, 128360. DOI: 10.1016/j.snb.2020.128360
5. H. Komatsu, Y. Shindo, K. Oka, J. P. Hill, K. Ariga, *Angew. Chem. Int. Ed.* **2014**, *53*, 3993-3995. DOI: 10.1002/anie.201311192
6. L. Wang, J. Zhang, B. Kim, J. Peng, S. N. Berry, Y. Ni, D. Su, J. Lee, L. Yuan, Y. T. Chang, *J. Am. Chem. Soc.* **2016**, *138*, 10394–10397. DOI: 10.1021/jacs.6b05810
7. M. A. Fomin, R. I. Dmitriev, J. Jenkins, D. B. Papkovsky, D. Heindl, B. König, *ACS Sens.* **2016**, *1*, 702–709. DOI: 10.1021/acssensors.5b00315
8. H. Chang, X. Hu, X. Tang, S. Tian, Y. Li, X. Lv, L. Shang, *ACS Sens.* **2023**, *8*, 829−838. DOI: 10.1021/acssensors.2c02458
9. L. Guan, W. Hu, H. Zuo, H. Sun, Y. Ai, M. He, C. Ma, M. Ding, Q. Liang, *Chem. Commun.* **2023**, *59*, 1617. DOI: 10.1039/d2cc06354a
